# Evaluation of Urban Local-Scale Aerodynamic Parameters: Implications for the Vertical Profile of Wind Speed and for Source Areas

**DOI:** 10.1007/s10546-017-0248-z

**Published:** 2017-04-28

**Authors:** Christoph W. Kent, Sue Grimmond, Janet Barlow, David Gatey, Simone Kotthaus, Fredrik Lindberg, Christos H. Halios

**Affiliations:** 1 Department of Meteorology, Reading University, RG6 6UR Reading, UK; 2 Risk Management Solutions, EC3R 8NB London, UK; 3 Department of Earth Sciences, University of Gothenburg, 405 30 Gothenburg, Sweden

**Keywords:** Aerodynamic roughness length, Anemometric methods, Logarithmic wind-speed profile, Morphometric methods, Source area, Zero-plane displacement

## Abstract

Nine methods to determine local-scale aerodynamic roughness length $(z_{0})$ and zero-plane displacement $(z_{d})$ are compared at three sites (within 60 m of each other) in London, UK. Methods include three anemometric (single-level high frequency observations), six morphometric (surface geometry) and one reference-based approach (look-up tables). A footprint model is used with the morphometric methods in an iterative procedure. The results are insensitive to the initial $z_{d}$ and $z_{0}$ estimates. Across the three sites, $z_{d}$ varies between 5 and 45 m depending upon the method used. Morphometric methods that incorporate roughness-element height variability agree better with anemometric methods, indicating $z_{d}$ is consistently greater than the local mean building height. Depending upon method and wind direction, $z_{0}$ varies between 0.1 and 5 m with morphometric $z_{0}$ consistently being 2–3 m larger than the anemometric $z_{0}$. No morphometric method consistently resembles the anemometric methods. Wind-speed profiles observed with Doppler lidar provide additional data with which to assess the methods. Locally determined roughness parameters are used to extrapolate wind-speed profiles to a height roughly 200 m above the canopy. Wind-speed profiles extrapolated based on morphometric methods that account for roughness-element height variability are most similar to observations. The extent of the modelled source area for measurements varies by up to a factor of three, depending upon the morphometric method used to determine $z_{d}$ and $z_{0}$.

## Introduction

The urban environment is arguably the most critical interface between humans and the atmosphere. Considerable progress has been made in understanding and modelling the urban environment across a broad spectrum of topics (e.g. Roth 2000; Arnfield 2003; Stewart 2011; Tominaga and Stathopoulos 2013). Wind speed is critical to the vertical and horizontal exchange of scalars and pollutants, and is important when considering, for example, the construction and insurance of buildings (Walker et al. 2016), pedestrian comfort (Stathopoulos 2006) and renewable energy (Drew et al. 2013). The world’s urban population is expected to increase to 66% by 2050 (UN 2014), and as cities grow outwards and more importantly upwards, larger populations become more exposed to urban wind regimes. Therefore, improved knowledge of urban flow effects is vital to the development of cities.

The prospect of an equilibrium boundary-layer wind-speed profile, represented using just a few parameters, is appealing, especially above a rough urban surface with complex flow across numerous length and time scales (Britter and Hanna 2003). Several relationships to describe the spatially- and temporally-averaged wind-speed profile above a surface exist, such as the power-law profile (Sedefian 1980), the logarithmic profile (Tennekes 1973) and profiles described by Deaves and Harris (1978), Emeis et al. (2007), Gryning et al. (2007) and Peña et al. (2010). A precursor to the use of each method is representation of the zero-plane displacement $(z_{d})$ and the aerodynamic roughness length $(z_{0})$.

Although the magnitude of both $z_{d}$ and $z_{0}$ is fundamentally related to surface morphology, assigning appropriate values remains challenging. This is particularly true in city centres, with pronounced variability in roughness-element heights and density, creating unique, complex surface morphology. Individual tall buildings often rise above mid-rise buildings, whilst in the suburbs more homogeneous roughness-element height and density are common.

The numerous methods used to determine $z_{d}$ and $z_{0}$ can be grouped into three classes: (i) reference-based, (ii) anemometric and (iii) morphometric. The reference-based method is the simplest, as a neighbourhood is compared to published tables or figures (e.g. Grimmond and Oke 1999; Wieringa et al. 2001; Stewart and Oke 2012) to determine appropriate values. Anemometric and morphometric methods both directly incorporate the unique surface morphology of an area and can account for variations in meteorological conditions (e.g. wind direction, wind speed or stability).

In the present study, high-quality databases are used to compare methods to determine $z_{d}$ and $z_{0}$ in urban areas. For the study area (central London, UK) the methods employed are: reference-based using aerial photography, anemometric using single- and multi-level observations and morphometric using digital elevation databases. Previous studies related to aerodynamic parameters relevant to London (Ratti et al. 2002, 2006; Padhra 2010; Drew et al. 2013; Kotthaus and Grimmond 2014b) have results that vary with the study area, method and gridded datasets (e.g. Evans 2009) used. Overall, the maximum $z_{d}$ and $z_{0}$ from these studies are 20 and 2 m, respectively. The objectives are a site-specific evaluation of: (i) the inter-method variability in aerodynamic parameters, and (ii) the implications for modelling the spatially- and temporally-averaged wind-speed profile.

The methodology to determine $z_{d}$ and $z_{0}$ through surface morphology is provided for use in the Urban Multi-scale Environmental Predictor (UMEP, http://www.urban-climate.net/umep/UMEP, Lindberg et al. 2016) for the open source geographical information software QGIS.

## Background

### The Urban Boundary Layer and Logarithmic Wind Law

The urban boundary layer is traditionally sub-divided into distinct layers (Fernando 2010), which are determined by urban surface characteristics and mesoscale conditions (Barlow 2014). Surface roughness elements are located within the urban canopy layer (UCL) (Roth 2000; Oke 2007), which experiences highly variable flow as a consequence of the close proximity to roughness elements. The UCL is within the roughness sublayer (RSL) (Roth 2000), of depth $H_{\textit{RSL}}$. The depth $H_{\textit{RSL}}$ is typically 2–5 times the average roughness-element height $(H_{\textit{av}})$ (Roth 2000; Barlow 2014), but can be considerably larger (e.g. Roth 2000, their Table 2), varying with the density (Raupach et al. 1991; Grimmond and Oke 1999; Roth 2000; Oke 2007; Barlow 2014), staggering (Cheng and Castro 2002) and height variability (Cheng and Castro 2002) of roughness elements, as well as meteorological conditions (Roth 2000). Idealized physical models (Cheng and Castro 2002; Kastner-Klein and Rotach 2004; Xie et al. 2008), large-eddy simulations (LES) (Giometto et al. 2016) and observations in a dense urban setting (Grimmond et al. 2004) suggest the minimum $H_{\textit{RSL}} =2H_{\textit{av}}$.

Between a height $z= H_{\textit{RSL}}$ and approximately 10% of the boundary-layer depth is the inertial sublayer (ISL), though when there is considerable roughness-element height variability the RSL encroaches upon the ISL (Cheng and Castro 2002; Cheng et al. 2007; Mohammad et al. 2015b) and an ISL may cease to exist (Rotach 1999). Within the ISL, the flow becomes free of the individual wakes and channelling associated with roughness elements, and the small variation of the turbulent fluxes of heat and momentum with height leads to the assumption of a constant-flux layer. In addition, if the airflow is fully adapted to upwind roughness elements (i.e. disregarding an internal boundary layer) a horizontally homogeneous flow is observed (Barlow 2014) and it is therefore possible to determine a spatially- and temporally-averaged wind-speed profile.

The logarithmic wind law applies in the ISL and during thermally neutral conditions can be used to estimate wind speeds to a height of approximately 200 m (Cook 1997) using surface-based length scales (i.e. $z_{d}$ and $\hbox {z}_{0}$) (Tennekes 1973),

1 $$\begin{aligned} \bar{u}_\mathrm{z}=\frac{u_{*}}{\kappa }\ln \left( \frac{z -{z}_{d}}{z_{0}}\right) , \end{aligned}$$

where $\bar{u}_\mathrm{z}$ is the mean horizontal wind speed at height *z*, $u_{*}$ is the friction velocity, and $\kappa =0.40$ is the von Karman constant (Högström 1996).

## Determination of Aerodynamic Parameters in Urban Areas

### Reference-Based Methods

Reference-based approaches require comparison between site photography and first-order height and/or density estimates to reference tables (e.g. Grimmond and Oke 1999; Wieringa et al. 2001). Wieringa’s (1993) comprehensive review of roughness length data provides tables for homogenous surfaces, whilst Grimmond and Oke (1999) focus upon urban areas, therefore the latter is used here.

### Morphometric Methods

#### Relations Between Aerodynamic Parameters and Roughness-Element Geometry

**Fig. 1:**
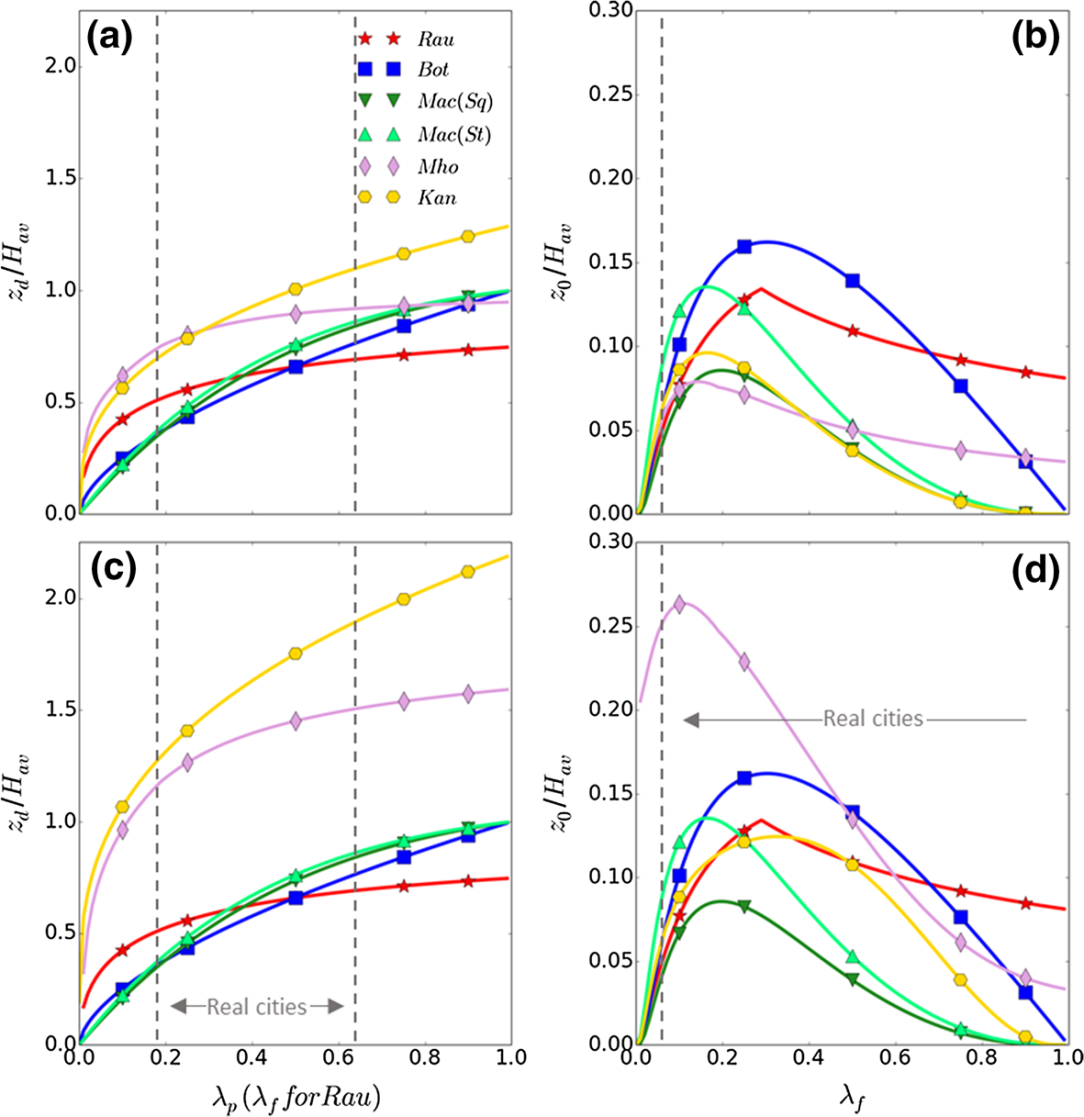
Zero-plane displacement $(z_{d})$ and aerodynamic roughness length $(z_{0})$ normalized by average roughness-element height $(H_{\textit{av}})$ for the morphometric methods assessed (Table 1), assuming roughness elements with: **a, b** homogeneous and **c, d** heterogeneous heights. Geometric parameters used are informed by the built surroundings within 1000 m of the KSSW site (Table 3): **a, b** $H_{\textit{av}}= 20\,\hbox {m}$, maximum height $(H_{\textit{max}})=20\,\hbox {m}$ and standard deviation of heights $(\sigma _{H})=0\,\hbox {m}$; **c, d** $H_{\textit{av}}=20\,\hbox {m}$, $H_{\textit{max}}=117\,\hbox {m}$, $\sigma _{H}=11\,\hbox {m}$. In both cases, frontal area index $(\lambda _{f})$ and plan area index $(\lambda _{p})$ are varied from 0 to 1, note $\lambda _{f}$ can become larger than 1. The *Mac* method is shown for square (*Sq*) and staggered (*St*) arrays. Real city limits are based on Grimmond and Oke (1999). *Curves* shown may extend beyond the extent to which the model was originally developed

Morphometrically-determined aerodynamic parameters in urban areas traditionally consider three flow regimes—isolated, wake interference and skimming (Oke 1987). These are related to the plan area index (ratio of plan built area occupied by roughness elements $(A_{\mathrm{p}})$ to total area under consideration $(A_{T}){:}\,\lambda _{p}= A_\mathrm{p}/A_{T})$ and frontal area index (ratio of the windward facing area of roughness elements $(A_{\mathrm{f}})$ to $A_{T}{:}\,\lambda _{f}= A_\mathrm{f}/A_{T}$). As surface cover $(A_\mathrm{p})$ increases the magnitude of $z_{d}$ scaled by $H_{\textit{av}}$ is traditionally observed to produce a convex curve asymptotically increasing from zero to 1 (Fig. 1a). In contrast, the relation between $\lambda _{f}$ and $z_{0}/H_{\textit{av}}$ has a peak at $\lambda _{f}$ between 0.1 and 0.4 depending on the method used to determine $z_{0}$ (Fig. 1b). The maximum possible $\lambda _{p}$ is unity, although $\lambda _{f}$ can exceed this.

Staggered and non-uniformly oriented groups of roughness elements generate a larger drag force than regular arrays, causing a more pronounced peak in $z_{0}$, as well as larger values of $z_{d}$ (Macdonald 2000; Cheng et al. 2007; Hagishima et al. 2009; Zaki et al. 2011; Claus et al. 2012). Roughness-element height variability also influences flow and turbulent characteristics, as the taller roughness elements generate a disproportionate amount of drag (Xie et al. 2008; Mohammad et al. 2015b). This suggests $z_{d}$ can be greater than the average roughness-element height (e.g. Jiang et al. 2008; Xie et al. 2008; Hagishima et al. 2009; Zaki et al. 2011; Millward-Hopkins et al. 2011; Tanaka et al. 2011; Kanda et al. 2013), with a peak $z_{0}$ up to five times greater and displaced to higher $\lambda _{f}$ (Hagishima et al. 2009; Zaki et al. 2011). Roughness-element staggering, orientation and most importantly height heterogeneity therefore need to be considered in morphometric calculations; especially in complex city centres, such as the current study site (Sect. 4.1).

#### Morphometric-Method Application in Urban Areas

Numerous morphometric methods exist (“Appendix”) and each method has its own assumptions and intended range of applicability. Newer methods have incorporated increasingly complex geometric features or theoretical ideas pertaining to the relation between aerodynamic parameters and surface morphology.

Here, six morphometric methods (Table 1) are selected for assessment that meet the following criteria: (i) both $z_{d}$ and $z_{0}$ are included in the formulations; (ii) the method is applicable to a wide range of urban densities and environments; (iii) geometric data required are readily obtainable in complex urban environments; (iv) given resources available, the method is computationally feasible. Hereafter, the methods assessed are referred to by their abbreviation in Table 1. When followed by subscript $z_{d}$ or $z_{0}$ the abbreviation refers to the zero-plane displacement or aerodynamic roughness length, respectively. The geometric parameters required by each method are shown in Table 1.

**Table 1:** Morphometric methods assessed (rows) with their required geometric parameters (columns)

Abbreviation	$H_{\textit{av}}$	$\lambda _{p}$	$\lambda _{f}$	$H_{\textit{max}}$	$\sigma _{H}$
Morphometric methods					
*RT*	$\checkmark$				
*Rau*	$\checkmark$		$\checkmark$		
*Bot*	$\checkmark$	$\checkmark$	$\checkmark$		
*Mac*	$\checkmark$	$\checkmark$	$\checkmark$		
*Mho*	$\checkmark$	$\checkmark$	$\checkmark$		$\checkmark$
*Kan*	$\checkmark$	$\checkmark$	$\checkmark$	$\checkmark$	$\checkmark$

Morphometric-method abbreviations: *RT* rule of thumb (Grimmond and Oke 1999), *Rau*  Raupach (1994), *Bot* Bottema and Mestayer (1998), *Mac*  Macdonald et al. (1998), *Mho* Millward-Hopkins et al. (2011), *Kan*  Kanda et al. (2013). Geometric parameters: $H_{\textit{av}}$ average roughness-element height, $\lambda _{p}$ plan area index, $\lambda _{f}$ frontal area index, $H_{\textit{max}}$ maximum roughness-element height, $\sigma _{H}$ standard deviation of roughness-element heights

The simplest, “rule of thumb” method (*RT*), only requires the average roughness-element height $(H_{\textit{av}})$ which is linearly related to $\textit{RT}_{{z}_{d}}$ and $\textit{RT}_{{z}_{0}}$,

2 $$\begin{aligned} \textit{RT}_{{z}_{d}}= & {} f_\mathrm{d}H_{\textit{av}}, \end{aligned}$$

3 $$\begin{aligned} \textit{RT}_{{z}_{0}}= & {} f_{0}H_{\textit{av}}, \end{aligned}$$

where the initial value used for $f_\mathrm{d}$ is 0.7 and for $f_{0}$ is 0.1 (Grimmond and Oke 1999). However, the value of $f_\mathrm{d}$ is revisited in Sect. 5.1.2.

Originally derived for vegetated surfaces, the Raupach (1994) method (*Rau*) provides reasonable results in urban environments (e.g. Bottema and Mestayer 1998; Grimmond and Oke 1999),

4 $$\begin{aligned} \textit{Rau}_{{z}_{d}}= & {} \left( 1+\left\{ \frac{\mathrm{exp}\left[ {-(C_{dl}2\lambda _{f})}^{0.5}-1 \right] }{{(C_{{ dl}}2\lambda _{f)}}^{0.5}} \right\} \right) H_{\textit{av}}, \end{aligned}$$

5 $$\begin{aligned} \textit{Rau}_{{z}_{0}}= & {} \left[ \left( 1-\frac{z_{d}}{H_{\textit{av}}} \right) \mathrm{exp}\left( -\kappa \frac{u_\mathrm{z}}{u_{*}}+{\varPsi }_{h} \right) \right] H_{\textit{av}}, \end{aligned}$$

with

6 $$\begin{aligned} \frac{u_{*}}{u_\mathrm{z}}=\mathrm{min}\left[ {{(C}_{\mathrm{S}}+C_{Dv}\lambda _\mathrm{f})}^{0.5},\left( \frac{u_{*}}{u_\mathrm{z}} \right) _{\mathrm{max}} \right] . \end{aligned}$$

Here $u_\mathrm{z}$ is the wind speed at roof height and empirical constants include: $C_{Dv}$ (the drag coefficient for vegetation $=0.3$), $C_{\mathrm{S}}$ (the drag coefficient for the substrate surface in the absence of roughness elements $= 0.003$), ${\varPsi }_{h}$ (the roughness-sublayer influence function—accounting for the correction to the logarithmic wind profile in the $\mathrm{RSL} =0.193),\,C_{dl}$ (a free parameter $= 7.5$) and $(u_{*}/u_\mathrm{z})_{\textit{max}}=0.3$. These constants suggested by Raupach (1994) are used here, but they do vary depending on roughness elements (Bottema and Mestayer 1998).

The Bottema and Mestayer (1998) method (*Bot*) is a simplified version of more complex formulations (Bottema 1995, 1997) specifically designed for urban areas. In the *Bot* method, a mutual sheltering parameter is used and it is assumed all of the drag experienced by the flow is due to roughness elements (therefore: $u_{*}=0.5\rho C_{{ Db}}u_\mathrm{z}^{2}\lambda _\mathrm{f}$, where $\rho$ is the density of air, and $C_{{ Db}}= 0.8$ is the drag coefficient for buildings),

7 $$\begin{aligned} \textit{Bot}_{{z}_{d}}= & {} \lambda _{p}^{0.6}H_{\textit{av}}, \end{aligned}$$

8 $$\begin{aligned} \textit{Bot}_{{z}_{0}}= & {} \left( z - z_{d} \right) \mathrm{exp}\left( \frac{\kappa }{\sqrt{0.5\lambda _{f}C_{{ Db}}} } \right) H_{\textit{av}}. \end{aligned}$$

The Macdonald et al. (1998) method (*Mac*) includes a fitting constant, $\alpha$, controlling the increase of $z_\mathrm{d}/H_{\textit{av}}$ with $\lambda _{p}$ and a drag correction coefficient $\beta$ to determine $z_{0}$,

9 $$\begin{aligned} \textit{Mac}_{{z}_{d}}= & {} \left[ 1+\alpha ^{-\lambda _\mathrm{p}}(\lambda _{p}-1) \right] H_{\textit{av}}, \end{aligned}$$

10 $$\begin{aligned} \textit{Mac}_{{z}_{0}}= & {} \left( \left( 1-\frac{z_\mathrm{d}}{H_{\textit{av}}}\right) \mathrm{exp}\left[ -\left\{ 0.5\beta \frac{C_{{ Db}}}{\kappa ^{2}}\left( 1-\frac{z_\mathrm{d}}{H_{\textit{av}}}\right) \lambda _\mathrm{f} \right\} ^{-0.5} \right] \right) H_{\textit{av}}. \end{aligned}$$

Macdonald et al. (1998) suggest $C_{{ Db}}=1.2$ and from wind-tunnel data (Hall et al. 1996) values of $\alpha =4.43$, $\beta =1.0$ for staggered arrays, and $\alpha =3.59$, $\beta =0.55$ for square arrays (Macdonald et al. 1998). The suitability of these experimental data as a fit to the constants has been questioned because of the short fetch used and lack of direct shear-stress measurement (Cheng et al. 2007). Ratti et al. (2002) propose a correction to the *Mac* method to account for roughness-element height variability $(z_{0} = \textit{Mac}_{{z}_{0}}[1+4(\sigma _{H}/H_{\textit{av}})]$, where $\sigma _{H}$ is the standard deviation of roughness-element heights). However, the correction is not considered here as no basis is provided and $z_{d}$ is not addressed. Kastner-Klein and Rotach’s (2004) empirically derived relationship using wind-tunnel results from a scaled physical model of Nantes, France, is also not considered because it does not incorporate $\lambda _\mathrm{f}$, a parameter that is regarded as important (Millward-Hopkins et al. 2011; Mohammad et al. 2015a).

Two morphometric methods that directly incorporate roughness-element height variability are explored: the *Mho* (Millward-Hopkins et al. 2011) and *Kan* (Kanda et al. 2013) methods. Both are yet to be independently evaluated. The *Mho* method describes the viscous drag associated with the unsheltered frontal area of roughness elements $(A^{*}_\mathrm{f})$ and their rooftops when density is below a critical threshold. The urban canopy is divided into layers and a cumulative-height normalized $z_{d}$ and drag balance is calculated. This process is computationally intensive and complex to operate (Tomlin, 2015, pers. comm.), therefore, a relation based on the more accessible standard deviation of roughness-element heights has been developed (Millward-Hopkins et al. 2011),

11 $$\begin{aligned} \textit{Mho}_{{z}_{d}}= & {} H_{\textit{av}}\left[ \frac{{\textit{Mho}U}_{{z}_{d}}}{H_{\textit{av}}}+\left( (0.2375 \ln \left( \lambda _\mathrm{p} \right) +1.1738)\frac{\sigma _{H}}{H_{\textit{av}}} \right) \right] , \end{aligned}$$

12 $$\begin{aligned} {\textit{Mho}}_{{z}_{0}}= & {} H_{\textit{av}}\left[ \frac{{\textit{Mho}U}_{{z}_{0}}}{H_{\textit{av}}}+\left( \mathrm{exp}\left( 0.8867\lambda _\mathrm{f} \right) -1 \right) \left( \frac{\sigma _{H}}{H_{\textit{av}}} \right) ^{\mathrm{exp}(2.3271\lambda _{f})} \right] , \end{aligned}$$

where

13 $$\begin{aligned} {\textit{Mho}U}_{{z}_{0}}= & {} \left( \left( 1-\frac{z_{d}}{H_{\textit{av}}} \right) \mathrm{exp}\left[ -\left\{ 0.5c_{{ Db}}\kappa ^{-2}\frac{A^{*}_\mathrm{f}}{A_{T}} \right\} ^{-0.5} \right] \right) H_{\textit{av}}, \end{aligned}$$

14 $$\begin{aligned} \frac{{\textit{Mho}U}_{{z}_{d}}}{H_{\textit{av}}}= & {} \left( \frac{19.2\lambda _{p}-1+\mathrm{exp}(-19.2\lambda _{p})}{19.2\lambda _{p}[1-\mathrm{exp}(-19.2\lambda _{p})]} \right) \left( \textit{for} \, \lambda _{p} \ge 0.19 \right) , \end{aligned}$$

15 $$\begin{aligned} \frac{{\textit{Mho}U}_{{z}_{d}}}{H_{\textit{av}}}= & {} \left( \frac{117\lambda _{p}+\left( 187.2\lambda _{p}^{3}-6.1 \right) \left[ 1-\exp \left( -19.2\lambda _{p} \right) \right] }{\left( 1+114\lambda _{p}+187\lambda _{p}^{3} \right) \left[ 1-\exp \left( -19.2\lambda _{p} \right) \right] } \right) \left( \textit{for} \, \lambda _{p}<0.19 \right) . \end{aligned}$$

The *Kan* method uses large-eddy simulations for real urban areas in Japan (107 grid squares of size 1000 m (*x*) by 1000 m (*y*) by 600 m (*z*) with a 2-m resolution) and 23 simple arrays from the literature (Cheng et al. 2007; Hagishima et al. 2009; Leonardi and Castro 2010; Zaki et al. 2011). Horizontally-averaged turbulent statistics, surface drag and wind-speed profiles were derived for each model grid and aerodynamic parameters determined through a least squares regression. Kanda et al. (2013) argue that the upper limit of $z_{d}$ is the maximum roughness-element height $(H_{\textit{max}})$, hence $H_{\textit{max}}$ is a more suitable scaling parameter than $H_{\textit{av}}$,

16 $$\begin{aligned} \textit{Kan}_{z_{d}}=\left[ c_{o}X^{2}+(a_{o}\lambda _{p}^{b_{o}}-c_{o})X \right] H_{\textit{max}}, \end{aligned}$$

where $a_{0}$, $b_{0}$ and $c_{0}$ are taken as 1.29, 0.36 and $-0.17$. *X* is the representative building height above the average building height $(\sigma _{H} + H_{\textit{av}})$, relative to the maximum building height,

17 $$\begin{aligned} X=\frac{\sigma _{H}+H_{\textit{av}}}{H_{\textit{max}}} , \end{aligned}$$

for $0\le X \le 1$. For $z_{0}$, the *Kan* method is a modification to $\textit{Mac}_{{z}_{0}}$,

18 $$\begin{aligned} \textit{Kan}_{z_{0}}=\left( b_{1}Y^{2}+c_{1}Y+a_{1} \right) \textit{Mac}_{{z}_{0}}, \end{aligned}$$

where $a_{1}$, $b_{1}$ and $c_{1}$ are empirically derived coefficients (0.71, 20.21 and $-0.77$), and *Y* accounts for the impact of $\lambda _{p}$ and $\sigma _{H}$ on $z_{0}$, tending to zero for homogeneous arrays (i.e. where $\sigma _{H}=0$),

19 $$\begin{aligned} Y = \frac{\lambda _{p} \sigma _{H}}{H_{\textit{av}}}, \end{aligned}$$

for $$0 \le Y$$.

The six morphometric methods are applied across a range of roughness-element densities with homogeneous (Fig. 1a, b) and heterogeneous (Fig. 1c, d) height. Their comparison demonstrates that aerodynamic parameters determined using the *RT*, *Rau*, *Bot* and *Mac* methods are independent of the height array used. Hereafter, these methods are collectively referred to as $\textit{RE}_{\textit{av}}$ (i.e. based upon average roughness-element height). In contrast, obvious differences occur for aerodynamic parameters determined using the *Mho* and *Kan* methods because of their direct consideration of height heterogeneity. Hereafter, the *Mho* and *Kan* methods are collectively referred to as $\textit{RE}_{\textit{var}}$ (i.e. they account for variable roughness-element heights).

Across the six methods, $z_{d}$ increases with $H_{\textit{av}}$ and $\lambda _{p}$ ($\lambda _{f}$ for $\textit{Rau}_{z_\mathrm{d}}$). The *Mho* and *Kan* methods both resolve the more considerable drag that is exerted by groups of roughness elements with height heterogeneity, therefore $\textit{Mho}_{z_\mathrm{d}}$ also increases with $\sigma _{H}$ and $\textit{Kan}_{z_{d}}$ increases with both $\sigma _{H}$ and $H_{\textit{max}}$. Results for $\textit{Bot}_{z_{d}}$ and $\textit{Mac}_{z_{d}}$ vary similarly with density $(\lambda _{p})$. The difference between $\textit{Mac}_{z_{d}}$ for square or staggered arrays is negligible compared to inter-method variability (Fig. 1a, c). For the homogeneous array (Fig. 1a, b) both $\textit{Kan}_{z_{d}}$ and $\textit{Mho}_{z_{d}}$ ($\textit{Mho}_{z_{d}}$ at $\lambda _{p}<0.8$) are larger than for the other morphometric methods. $\textit{Kan}_{z_{d}}$ becomes larger than $H_{\textit{av}}$ and $\textit{Mho}_{z_{d}}$ levels off, implying both do not fulfil the requirement that $z_\mathrm{d}/H_{\textit{av}}=1$ when $\lambda _{p}=1$. Therefore, when $\lambda _{p}>0.50$ the *Kan* and *Mho* methods may under- and over-estimate $z_{d}$ for homogeneous arrays, respectively. As the methods were derived from datasets with 0.05 $<\lambda _{p} <0.50$ this is beyond their limits, and is uncommon for real cities (e.g. Fig. 1).

When roughness-element height heterogeneity is introduced (Fig. 1c, d), the $\textit{RE}_{\textit{av}}$ method results are identical to the homogeneous case because $H_{\textit{av}}$ is the only height attribute used. In fact, $\textit{Kan}_{z_{d}}$ and $\textit{Mho}_{z_{d}}$ increase by a factor of approximately two and are therefore consistently twice the values for the $\textit{RE}_{\textit{av}}$ methods. The increase of $\textit{Kan}_{z_{d}}$ and $\textit{Mho}_{z_{d}}$ suggests $z_{d}$ is larger than $H_{\textit{av}}$ for most plan area densities. This is especially true for $\textit{Kan}_{z_{d}}$, which scales with $H_{\textit{max}}~$(assumed 117 m) and increases with density to become over twice $H_{\textit{av}}$.

For each method, $z_{0}$ increases to a maximum ‘critical’ frontal area index $(\lambda _{{f}{} { -crit}})$, and when roughness elements have homogeneous heights (Fig. 1b), $\lambda _{{f}{\textit{-crit}}}$ varies from a minimum of $0.11\,(\textit{Mho}_{z_{0}})$ to a maximum of 0.3 $(\textit{Bot}_{z_{0}})$. The peak magnitude is similar for $\textit{Mac}_{z_{0}}$ for square arrays, $\textit{Mho}_{z_{0}}$ and $\textit{Kan}_{z_{0}}\,(0.1 H_{\textit{av}})$, which is smaller than $\textit{Mac}_{z_{0}}$ for staggered arrays, $\textit{Rau}_{z_{0}}$ and $\textit{Bot}_{z_{0}}\,(0.15H_{\textit{av}})$. The decrease in $z_{0}$ beyond $\lambda _{{f}{\textit{-crit}}}$ is most obvious for $\textit{Mac}_{z_{0}}$, whilst $\textit{Bot}_{z_{0}}$ remains larger across its wider peak. When height heterogeneity $(\sigma _{H})$ is introduced (Fig. 1d), an increase in $\textit{Kan}_{z_{0}}$ and especially $\textit{Mho}_{z_{0}}$ (up to a factor of four) is a response to the additional drag imposed by roughness elements of variable heights (Eqs. 12, 18). The $\textit{Kan}_{z_{0}}$ peak broadens to cover a wider range of densities.

### Anemometric Methods

Multiple anemometric methods exist (“Appendix”) that use slow and fast response sensors located at appropriate heights for which the logarithmic wind law is valid (Sect. 2.2). As single-level observations are more frequently available, two methods to determine $z_{d}$ and one to determine $z_{0}$ from single level, high frequency measurements are assessed. These use the meteorological variables indicated in Table 2.

**Table 2:** Anemometric methods used to calculate the **(a)** zero-plane displacement $(z_{d})$ and **(b)** aerodynamic roughness length $(z_{0})$ with their respective meteorological variables and required stability condition

Abbreviation	*z*	$z_{d}$	*L*	$u_{*}$	$\bar{u}_\mathrm{z}$	$\sigma _\mathrm{w}$	$\sigma _\mathrm{u}$	$\sigma _{T}$	$T_{*}$	Stability
Anemometric methods										
(a) $$z_{d}$$										
TVM	$\checkmark$		$\checkmark$					$\checkmark$	$\checkmark$	Unstable
WVM	$\checkmark$		$\checkmark$	$\checkmark$		$\checkmark$				Unstable
(b) $$z_{0}$$										
EC	$\checkmark$	$\checkmark$	$\checkmark$	$\checkmark$	$\checkmark$					Neutral

Methods *TVM* temperature variance method (Rotach 1994), *WVM* wind variance method (Toda and Sugita 2003), *EC* eddy covariance method (Grimmond et al. 1998). Variables *z* measurement height, $z_\mathrm{d}$ zero-plane displacement, *L* Obukhov length, $u_{*}$ friction velocity, $\bar{u}_\mathrm{z}$ mean horizontal wind speed at height $z,\,\sigma _\mathrm{w}$ standard deviation of vertical velocity, $\sigma _\mathrm{u}$ standard deviation of horizontal velocity component, $\sigma _{T}$ standard deviation of temperature, $T_{*}$ temperature scale

To determine $z_{d}$ the ‘temperature variance’ (Rotach 1994, Eq. 20) and ‘wind variance’ (Toda and Sugita 2003, Eq. 21) methods are used. These methods, based upon surface-layer scaling (Monin-Obukhov similarity theory), use the relation between the non-dimensional temperature variance, vertical velocity variance, and stability parameter *z* / *L* (Wyngaard et al. 1971; Tillman 1972),

20 $$\begin{aligned} \phi _{T}= & {} \frac{\sigma _{T}}{T_{*}}= {-C}_{1}\left( C_{2}-\frac{z -{ z}_{d}}{L} \right) ^{-\frac{1}{3}} , \end{aligned}$$

21 $$\begin{aligned} \phi _\mathrm{w}= & {} \frac{\sigma _\mathrm{w}}{u_{*}}= C_{3}\left( 1-C_{4}\left[ \frac{z - z_{d}}{L} \right] \right) ^{\frac{1}{3}}, \end{aligned}$$

where $\sigma _{T}$ and $\sigma _\mathrm{w}$ are the standard deviation of temperature and vertical velocity respectively, $T_{*}$ is the temperature scale $(T_{*}=-\left( \overline{w^{'}T^{'}} \right) /u_{*})$, *L* is the Obukhov length $(L=-\bar{T}u_{*}^{2}\big /\kappa g T_{*}$, with *g* the acceleration due to gravity). Constants $C_{1}$ to $C_{4}$ are derived from observations, which vary across experiments and surfaces (e.g. Sorbjan 1989; Hsieh et al. 1996; Roth 2000). Using constants where $z_\mathrm{d}$ is assumed negligible ($C_{1}=0.99$, $C_{2}=0.06$, $C_{3}=1.25$ and $C_{4}=3$, Toda and Sugita 2003), the differences between observed $(\phi _{\textit{obs}})$ and estimated $(\phi _{\mathrm{est}})~\phi _{T}$ and $\phi _\mathrm{w}$ are compared. The $z_{d}$ is incrementally increased providing a new $\phi _{\mathrm{est}}$ value (for *n* iterations) and the $z_{d}$ value, which minimizes the root-mean-square error (*RMSE*) is taken as the appropriate value of $z_{d}$,

22 $$\begin{aligned} \textit{RMSE}=\sqrt{\left( \frac{1}{n} \right) \sum \limits _{i=1}^n \left[ (\phi _{est})-(\phi _{obs}) \right] ^{2} } . \end{aligned}$$

With $z_{d}$ determined and a direct observation of $u_{*}$, the eddy-covariance (EC) method allows calculation of $z_{0}$, through rearrangement of the logarithmic wind law,

23 $$\begin{aligned} z_{0}=(z - z_{d})~\mathrm{exp}\left( -\frac{\bar{u}_\mathrm{z}\kappa }{u_{*}} \right) , \end{aligned}$$

where $\bar{u}_\mathrm{z}$ and $u_{*}$ are determined from observations at *z*. The EC method is applicable during near-neutral stability if stationarity is met (Foken and Wichura 1996). At least 20 observations are required to determine $z_{0}$ for a given wind-direction sector (Beljaars 1987; Grimmond et al. 1998). In addition, low wind speeds $(\bar{u}_\mathrm{z}<1~\mathrm{m } \mathrm{s}^{-1})$ are excluded (Liu et al. 2009).

## Methods

### Site Description

**Fig. 2:**
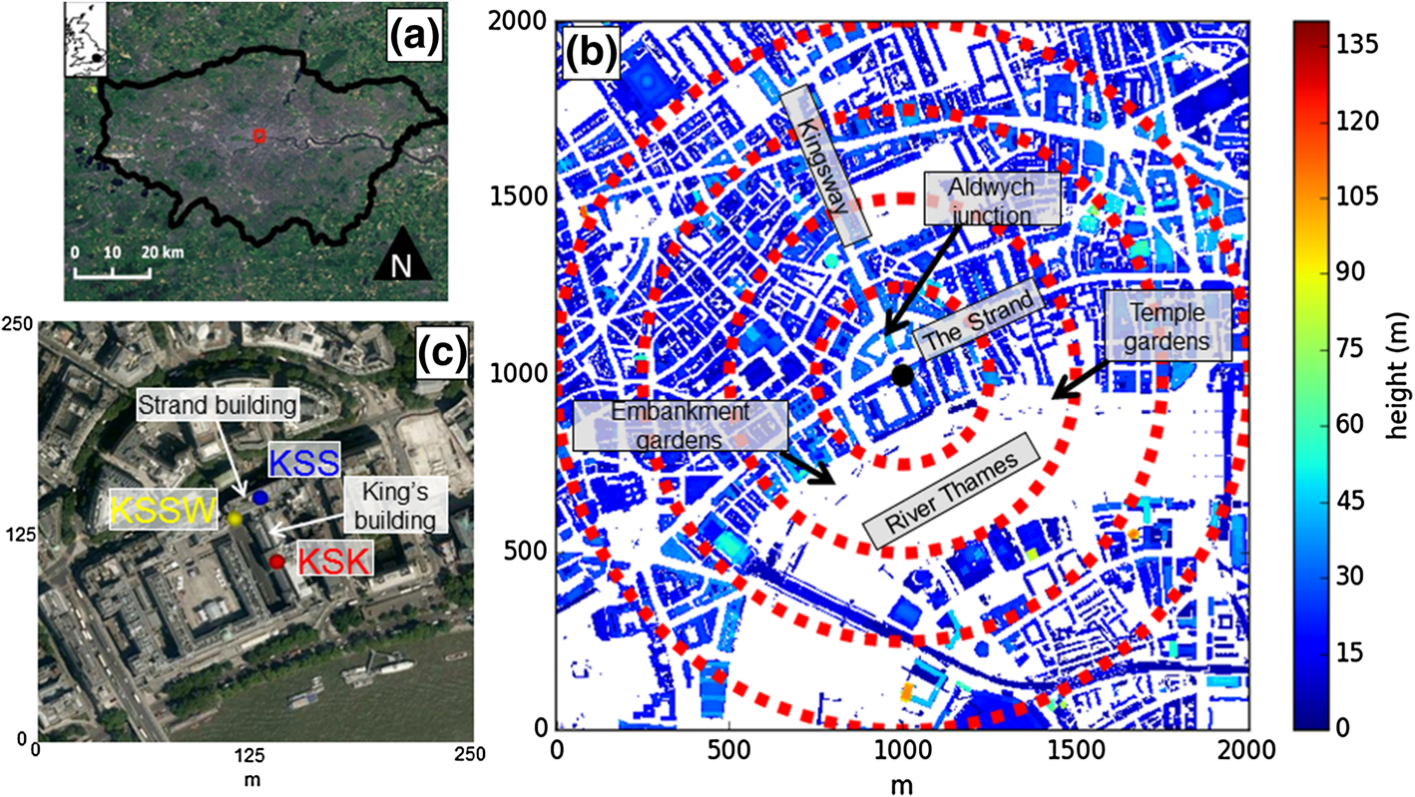
Location of measurement sites KSK, KSS and KSSW at King’s College London (KCL) (see text for details) **a** within Greater London, UK (*inset*); **b** building heights surrounding the sites (major features labelled), *red dashed lines* indicate 250, 500, 750 and 1000-m radii from the KSSW site (*black point*); and **c** 250 m surroundings of KCL. *Photography source*: Google imagery (2014)

Three London Urban Meteorological Observatory network (http://micromet.reading.ac.uk/) sites in the central activities zone of London (Fig. 2a) are used, where prior analyses have been undertaken (e.g. Kotthaus and Grimmond 2014a, b; Björkegren et al. 2015; Ward et al. 2015). Instrumentation at the Strand campus of King’s College London (KCL) has been mounted on towers upon the King’s building (KSK), the Strand building (KSS), and to the west on the Strand building (KSSW) (Fig. 2c). The sites are all within 60 m, so their surroundings are similar. The local climate zone (Stewart and Oke 2012) ‘compact midrise’, is characterized by taller buildings amidst midrise building stock. Land cover is mostly paved and buildings constructed with stone, brick, tile, and concrete. Small gardens are located approximately 200 m east and 250 m south-west of the sites (Fig. 2b), with larger expanses of vegetation in parks over 1 km to the west of the sites. Street canyons are located immediately north of the KSS and KSSW sites. One canyon (The Strand) extends for over 1 km in the north-east to south-west directions (orientation: $060^{\circ }$–$240^\circ$), and another (Kingsway) extends approximately 500 m to the north-north-west (orientation: $330^\circ$) (Fig. 2c). The River Thames is located to the south between directions $092^{\circ }$–$223^\circ$ (site dependent, Table 3). Although geometric parameters and land cover vary with direction and meteorological conditions (through the measurement source area, Sect. 7), values based on a 1-km radius are provided in Table 3.

### Observations

The period analysed for aerodynamic parameter determination is 2014 for the KSSW site and 2011 for the KSS and KSK sites. During independent assessment of the methods (Sect. 6), an additional 2 months in 2010 are considered at the KSS site. Identical instrumentation is used at the KSS and KSSW sites, as the equipment was moved along the Strand building (Fig. 2c) in 2012 preventing temporal overlap across all sites. The periods analyzed allow for seasonal variability of meteorological conditions, whilst limiting surface cover changes (e.g. construction).

A sonic anemometer (CSAT3, Campbell Scientific, US) measured the three-dimensional wind velocity and sonic temperature at a sampling frequency of 10 Hz at each site. The anemometers were supported by a single tube mast at the KSK site (Clark Masts CSQ T97/HP) and a triangular tower at the KSSW and KSS sites (Aluma T45-H). Instrument orientation was south-westerly to minimize potential mast-induced distortion for the prevailing wind directions.

The sensor heights are at $z=1.97H_{\textit{av}}$ (KSK), $2.48H_{\textit{av}}$ (KSS) and $2.55H_{\textit{av}}$ (KSSW) for $H_{\textit{av}}$ in the surrounding area of 1-km radius (Table 3). Although relative heights vary with direction and meteorological conditions (e.g. Sect. 7), measurements at the KSK site are closest to the top of the RSL and therefore more likely to be affected by roughness-element wakes. In contrast the sensors at the KSS and KSSW sites are assumed to be at heights above the RSL. To evaluate this assumption, analysis of drag coefficient and turbulence intensities was undertaken around the sites to identify potential flow disturbance from nearby roughness-element wakes (e.g. Barlow et al. 2009). The analysis at the KSK site reveals that flow from the northern sector is disturbed by the Strand building (Fig. 2c, as noted by Kotthaus and Grimmond 2014b). At the KSS site, disturbance of flow is aligned with a nearby rooftop microscale anthropogenic source of moisture and heat that has previously been shown to influence turbulent fluxes (Kotthaus and Grimmond 2012). At the KSSW site, potential disturbance is aligned with a tall slender structure protruding from the Strand building roof (Fig. 2c). Elsewhere, no disturbance is identified, indicating the measurements at the KSS and KSSW sites are predominantly clear of roughness-element wakes and therefore above $z=H_{\textit{RSL}}$.

Data are pre-processed following Kotthaus and Grimmond (2014a). Eddy-covariance planar fit coordinate transformation is performed using ‘ECpack’ software (Van Dijk et al. 2004) and a yaw rotation provides wind speed aligned to the mean direction (Kaimal and Finnigan 1994). Humidity corrections are applied to the sonic temperature (Schotanus et al. 1983) and 30-min flux calculations are used to capture both the high and low end of the energy spectrum. An Ogive test (Moncrieff et al. 2004) ensured that this was an appropriate time period.

A Halo Photonics Streamline pulsed Doppler lidar situated at the KSSW site for 8 months (Table 3) operated in Doppler beam swinging mode, as outlined by Lane et al. (2013). The lidar, measuring wind speed and direction, has 30-m gates with the mid-point of the first usable gate 141 m above ground level. The sampling interval of 120 s allows 1-h averages to be calculated, which reduces error in the mean wind speed, whilst also ensuring stationarity (Lane et al. 2013).

**Table 3:** Characteristics of the measurement sites within a 1-km fetch: **(a)** sensor heights: metres above ground level, river position: bearing of the most northern point of the north bank, **(b)** geometric parameters and **(c)** surface cover

Site	WGS84: Lat (N), Lon (E)	Instrument	Sensor height (m a.g.l.)	Host roof height (m)	Observation period analysed	Potential flow disturbance (bearing $^{\circ })$	River position (bearing $^{\circ })$	
(a) Instrument locations								
KSSW	$51^\circ 30^{\prime }42.48^{\prime \prime }$–$0^\circ 7^{\prime }0.192^{\prime \prime }$	Halo Photonics Streamline pulsed Doppler lidar	–	35.6	1 Oct 2010–18 May 2011	–	097–212	
			50.3	35.6	1 Jan 2014–31 Dec 2014	230–245		
KSS	$51^\circ 30^{\prime }43.2^{\prime \prime }$–$0^\circ 6^{\prime }58.8594^{\prime \prime }$	CSAT3 Campbell Scientific 3D sonic anemometer	48.9	35.6	1 Oct 2010–31 Dec 2011	045–090	095–215	
KSK	$51^\circ 30^{\prime }41.04^{\prime \prime }$–$0^\circ 6^{\prime }57.9594^{\prime \prime }$		38.8	30.2	1 Jan 2011–31 Dec 2011	270–045	092–223	

$H_{\textit{av}}$ (m)	$\lambda _{p}$	$H_{\textit{max}}$ (m)	$\sigma _{H}$ (m)	Built	Paved	Grass	Trees and shrubs	Water
(b) Geometric parameters				(c) Surface cover (%)				
19.74	0.41	116.72	10.83	40.7	40.3	6.8	1.00	11.2

### Determination of Aerodynamic Parameters

#### Flow Diagram Illustrating Framework of Analysis

**Fig. 3:**
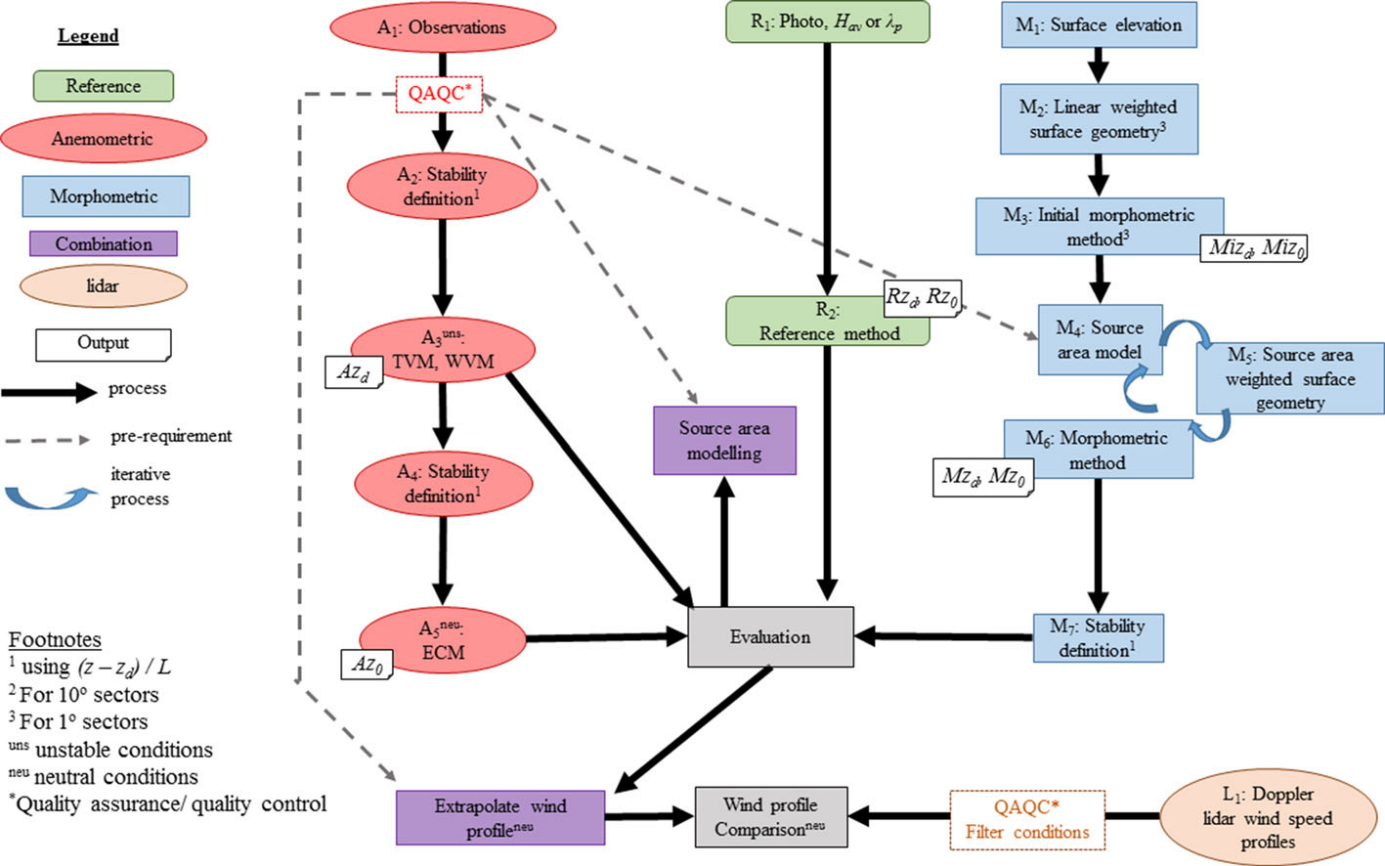
Flow diagram of the determination of aerodynamic parameters at the London sites using anemometric (*A*), reference (*R*) and morphometric (*M*) methods

At each of the measurement sites, local aerodynamic parameters are determined using the reference-based, morphometric and anemometric methods (Fig. 3) and evaluated (Sect. 5). Wind-speed profiles are then extrapolated using the logarithmic wind law (Eq. 1) and aerodynamic parameters from each method for comparison to wind speeds observed aloft using Doppler lidar (Fig. 3, $\hbox {L}_{\mathrm{1}}$) (Sect. 6). An example of the impacts upon the source area for measurements is also shown (Sect. 7).

Application of the reference-based approach only requires aerial photography (Fig. 3, $R_{\mathrm{1}}$) to provide aerodynamic parameters (Fig. 3, $R_{\mathrm{2}}$). The more involved anemometric and morphometric determination of $z_{d}$ and $z_{0}$ are expanded upon in Sects. 4.3.2 and 4.3.3, respectively. Decisions or available resources at each step potentially influence results; e.g. if a source area footprint model is used (Fig. 3, $\hbox {M}_{{4}}$).

#### Anemometric Determination of Aerodynamic Parameters

To determine $z_{d}$ with the temperature and velocity variance methods (Fig. 3, $\hbox {A}_{\mathrm{3}})$, $10^\circ$ directional sectors are used ($000^{\circ }{-}010^{\circ }$, etc) to provide sufficient observations whilst allowing for varying fetch. As the methods require unstable conditions ($0.05 \le z'/L \le 6.2$, Roth 2000, where $z' = z-z_{d}$), an a priori assumption of $z_{d}$ is required (Fig. 3, $\hbox {A}_{\mathrm{2}})$. The methods are applied by defining stability with several values of $z_{d}$, ranging from zero to the measurement height in 5-m increments, providing a range of solutions for each $10^{\circ }$ sector. If the denominator in $\phi _{T}$ $(T_{*})$ or $\phi _\mathrm{w} (u_{*})$ (Eqs. 20, 21, respectively) approaches zero, periods are removed. The additional criteria of $u_{*}>0.05\hbox { m s}^{-1}$ and $T_{*}<-0.05\hbox { K}$ may remove difficulties encountered using the methods in previous studies (e.g. De Bruin and Verhoef 1999; Rooney 2001). The methods are applied using rural ($C_{1}-C_{4}$, Sect. 3.3) and urban (Roth 2000) constants, as well as those determined using non-linear regression (Bates and Watts 1988) of Eqs. 20 and 21 to observations at each site. However, the two latter methods require an a priori assumption of $z_{d}$ and therefore provide a solution that is similar to the initial $z_{d}$, and not useful.

The $z_{d}$ value from both the temperature and velocity variance methods for each $10^{\circ }$ sector are used to determine neutral conditions $|z'/L|\le 0.05$ (Fig. 3, $\hbox {A}_{\mathrm{4}})$, and subsequently to calculate $z_{0}$ (Fig. 3, $\hbox {A}_{\mathrm{5}})$ using the EC method (Eq. 23).

#### Morphometric Determination of Aerodynamic Parameters

A 4-m resolution surface elevation dataset (Lindberg and Grimmond 2011) is used to determine the geometric parameters required to apply the morphometric methods (Fig. 3, $\hbox {M}_{{1}}$). For each morphometric method an initial estimation of $z_{d}$ and $z_{0}$ is made for $1^{\circ }$ sectors and a 1-km fetch ($\textit{Miz}_{d}$, $\textit{Miz}_{0}$) (Fig. 3, $\hbox {M}_{{3}}$). During this process, four annuli are used (0–250, 250–500, 500–750 and 750–1000 m; e.g. Fig. 2b for the KSSW site) to weight surface geometry (50.00, 31.25, 12.5 and 6.25%, respectively), based on Kotthaus and Grimmond’s (2014b) footprint climatology. The Kormann and Meixner (2001) analytical footprint model (Fig. 3, $\hbox {M}_{{4}}$) is then used to indicate the probable extent of the turbulent flux source area for each 30-min period of meteorological observations. The footprint model requires the measurement height and the observed $\sigma _{v}$ (standard deviation of the lateral velocity component), $\textit{L},\,u_{*}$ and wind direction. It also requires $z_{d}$ and $z_{0}$, hence their initial estimation ($\textit{Miz}_{d}$ and $\textit{Miz}_{0}$) that is averaged across $\sigma _{v}$ for each period of observations (Kotthaus and Grimmond 2014b).

The 80% cumulative source area for each measurement (30-min) is used to weight the fractional contribution of each grid square in the surface elevation database (Fig. 3, $\hbox {M}_{\mathrm{5}}$). A weighted geometry is then determined, allowing for source area specific aerodynamic parameters ($\textit{Mz}_{d}$ and $\textit{Mz}_{0}$) to be calculated for each morphometric method (Fig. 3, $\hbox {M}_{\mathrm{6}})$. The $\textit{Mz}_{d}$ and $\textit{Mz}_{0}$ values for each observation period are iteratively provided to the source area model until the mean absolute difference of the parameter between iterations is $<5{\%}$ or four iterations are performed. The latter is deemed appropriate given computational requirements and the range of values across the methods (Sect. 5). The methodology implies that $\textit{Mz}_{d}$ and $\textit{Mz}_{0}$ vary for each 30-min time period as a consequence of the varying source area. When the source area becomes so small that it covers only the nearest few roughness elements (e.g. during very unstable conditions or large $z_{d}$) a morphometrically determined $z_{d}$ or $z_{0}$ is inappropriate. Therefore, only source areas extending horizontally beyond 100 m from the measurement sensor are considered.

The initially-estimated aerodynamic parameters (Fig. 3, step $\hbox {M}_{{3}}$: $\textit{Miz}_{d}$ and $\textit{Miz}_{0}$) were found to be independent of the solution, irrespective of source area model (Kormann and Meixner 2001; Kljun et al. 2015 models used). Thus, it is possible to omit steps $\hbox {M}_{\mathrm{2}}$ and $\hbox {M}_{\mathrm{3}}$ (Fig. 3) and initialize the model with any reasonable roughness parameters (e.g. open country: $z_{0}= 0.03\,\hbox {m},\,z_{d}= 0.2\,\hbox {m}$). Here, steps $\hbox {M}_{{2}}$ and $\hbox {M}_{{3}}$ are retained for completeness. In addition, the Kormann and Meixner (2001) model is used, as the Kljun et al. (2015) model requires specification of the boundary-layer height, which is not available for all observations.

## Results

### Zero-Plane Displacement $(z_{d})$

#### $z_{d}$ Determined by Anemometric Methods

**Fig. 4:**
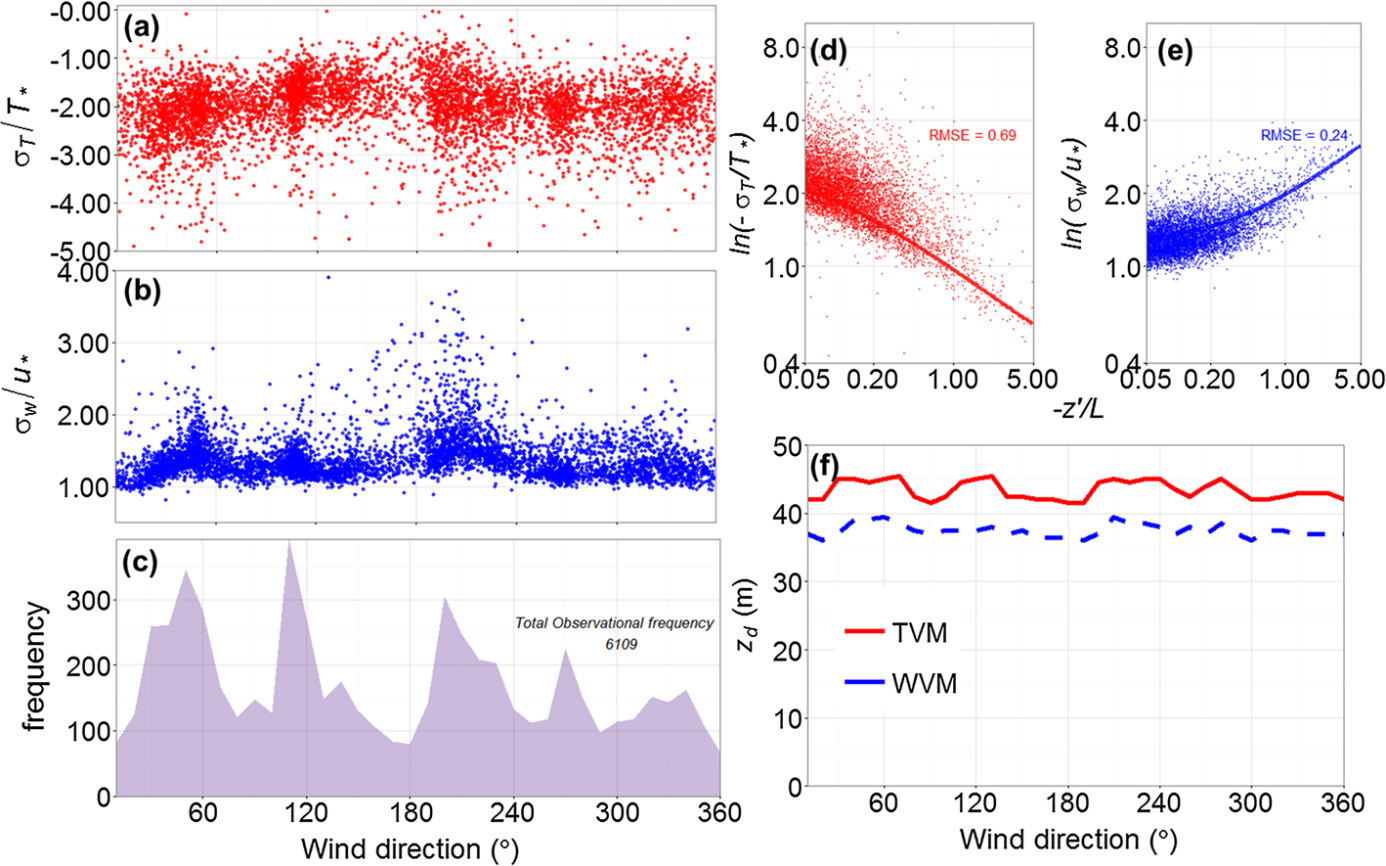
Application of the temperature variance (TVM) and wind variance (WVM) anemometric methods at the KSSW site to determine $z_{d}$ during unstable conditions $(-6.2 \le z'/L \le -0.05)$, with $z_{d}=30\,\hbox {m}$ used to define stability. Results shown are 30-min observations (*points*) of the scaled: **a** standard deviation of temperature $(\sigma _{T}/T_{*})$ and **b** vertical wind velocity $(\sigma _\mathrm{w}/u_{*})$ by wind direction; and **c** frequency of unstable conditions for $10^{\circ }$ bins. Non-linear fit (*line*) to observations for **d** TVM (Eq. 20) and **e** WVM (Eq. 21), with *RMSE* values; **f** Solution for $z_{d}$ ($10^{\circ }$ sectors) for the TVM (*red solid line*) and WVM (*blue dashed line*) methods

**Fig. 5:**
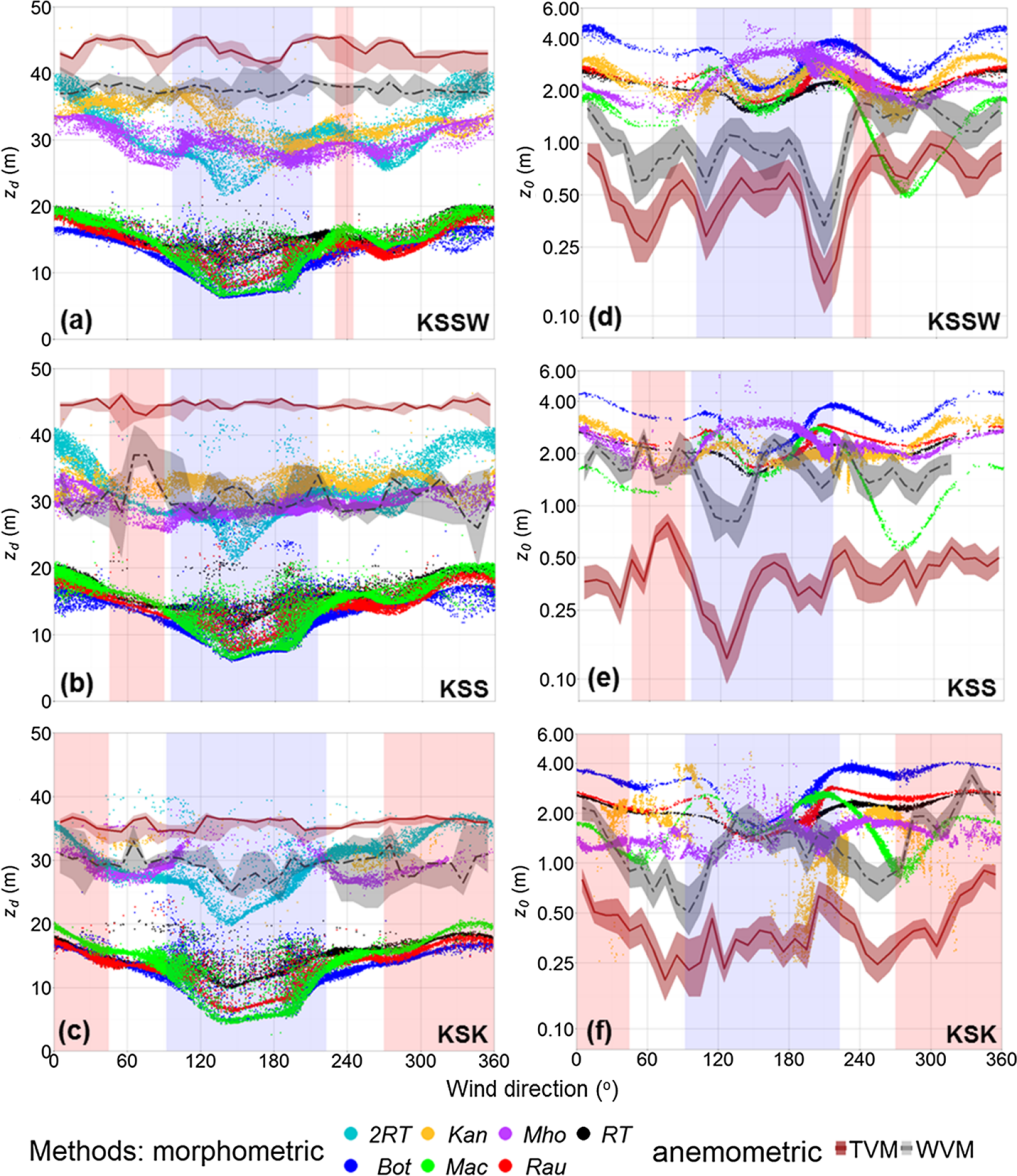
Comparison of anemometric (*lines* and *shading*) and morphometric (*points*) methods to determine the **a, b, c** zero-plane displacement $(z_{d})$ and **d, e, f** aerodynamic roughness length $(z_{0})$ (note ln *y* axis) surrounding the three assessed sites (Fig. 2). For anemometric methods, $z_{d}$ is the median solution of the temperature variance (TVM, *solid line*) and velocity variance (WVM, *dashed line*) methods, respectively, applied to 30-min observations during unstable conditions $(-6.2 \le z'/L \le -0.05)$ for $10^\circ$ sectors. The range (*shading*) represents all possible solutions by varying $z_{d}$ used for stability definition from zero to the measurement height; $z_{0}$ is the median (*lines*) and upper and lower quartile (*shaded*) of the eddy-covariance method (Eq. 23) during neutral conditions $(|z'/L|\le 0.05)$ for each $10^{\circ }$ sector using $z_{d}$ from the TVM and WVM, respectively. Morphometric methods use geometry weighted by the final iterated footprint for each 30-min observation (Sect. 4.3.3) for the same stability conditions as anemometric methods, however, $z_{d}$ in $z'/L$ is determined by the respective morphometric method for each observation. *Background shading* is in directions where the River Thames is located (*blue*) and where turbulence data indicate a disturbance (*red*). For method abbreviations see Tables 1 and 2

The stages of the application of the temperature and velocity variance methods are demonstrated for the KSSW site in Fig. 4. The $z_{d}$ values determined by each method are unbiased by the initial $z_{d}$ used to define stability (Sect. 4.3.2), which causes <5-m variability in any wind direction (indicated by the range in each method, Fig. 5). In addition, the impact of varying the empirical coefficients $C_{1}-C_{4}$ (Sect. 3.3.) (based on Sorbjan 1989 and Hsieh et al. 1996) is $<5\,\hbox {m}$ in any $10^{\circ }$ sector, and therefore generates similar uncertainty to that of the stability definition (Fig. 5a–c).

The similarity relations (Eqs. 20, 21) for temperature are consistently associated with a larger *RMSE* value compared to those for vertical velocity (e.g. Fig. 4d, e), because the temperature data have a relatively larger spread. Across sites, *RMSE* values for the velocity variance method relation varies between 0.18 and 0.49, whilst it is 0.35–0.97 for the temperature variance method. The larger *RMSE* values associated with the temperature data may be caused by the thermal inhomogeneity of the area. The *RMSE* value for the temperature data increases with height (i.e. the largest *RMSE* value is observed at the KSSW site), which is attributable to the larger extent of the source area and more numerous sources and sinks of heat.

Toda and Sugita (2003) suggest application of both the temperature and velocity variance methods assist in the determination of $z_{d}$. This is true at both the KSSW and KSK sites where $z_{d}$ determined using each method varies by approximately 5 m for each $10^{\circ }$ sector (Figs. 4f,  5a, c). In comparison, the method solutions at the KSS site consistently vary by $>13\,\hbox {m}$ (Fig. 5b). The large variability at the KSS site is most likely associated with the nearby rooftop microscale anthropogenic sources of moisture and heat (Kotthaus and Grimmond 2012) influencing turbulent fluxes.

The $z_{d}$ based on the temperature variance method is consistently larger than that for the velocity variance method (Fig. 5a–c). Previous studies found $z_{d}$ may be larger than $H_{\textit{av}}$ in urban areas using both the temperature (Grimmond et al. 1998, 2002; Feigenwinter et al. 1999; Kanda et al. 2002; Christen 2005; Chang and Huynh 2007; Tanaka et al. 2011) and velocity (Tsuang et al. 2003) variance approaches. Results at the KCL sites support this, as $z_{d}$ is up to twice $H_{\textit{av}}~(H_{\textit{av}}= 19.74\,\hbox {m}$, Table 3).

No obvious association is evident between the directional variability of $z_{d}$ and surface characteristics. For the temperature variance method, $z_{d}$ is similar for all directions at each site (Fig. 5a–c), varying by < 5 m. Whereas, the velocity variance method $z_{d}$ varies by up to 10 m, possibly because of occasional flow interference from roughness-element wakes. The parks (1–2 km upwind to the west) do not obviously influence $z_{d}$, but considering the extent of the source area for the measurements (Sect. 7) this is expected. The River Thames (Fig. 5a–c, blue shading) and small parks (Fig. 2b) closer to the measurement sites also do not affect the $z_{d}$ values. Following Jackson (1981), $z_{d}$ is the centroid of the drag profile of the roughness elements. The lack of directional variability in anemometric $z_\mathrm{d}$ indicates the surface drag is dominated by taller roughness elements (maximum building height is 40–60 m in all directions). This is consistent with the disproportionate amount of drag observed to be exerted by taller roughness elements in a heterogeneous mix (Xie et al. 2008; Mohammad et al. 2015b).

#### $z_{d}$ Determined by Morphometric Methods

There is less inter-site variability in $z_{d}$ values determined using each morphometric method, compared to the anemometric methods (Fig. 5a–c). However, the range of values between morphometric methods (intra-site variability) is larger than for the anemometric methods. There is an obvious separation between the methods based upon uniform (*RT*, *Bot*, *Rau*, *Mac*: $\textit{RE}_{\textit{av}})$ and heterogeneous (*Kan* and *Mho*: $\textit{RE}_\mathrm{var})$ roughness-element heights. Across the sites, the former range between 5 and 20 m, whereas the latter are between 25 and 40 m (or almost twice the $\textit{RE}_{\textit{av}}$ methods). The river, between directions $092^{\circ }{-}223^{\circ }$ (site dependent, see Table 1), causes a reduction in average height and therefore also in $z_{d}$ determined by the $\textit{RE}_{\textit{av}}$ methods. In comparison, the $\textit{RE}_{\textit{var}}$ methods are unresponsive because $\sigma _{H}$ becomes larger in these directions. The variability between the morphometric methods therefore becomes at least a factor of four in directions where the river is located.

When the measurement footprint has higher urban densities (non-river directions) $z_{d}$ determined by the $\textit{RE}_{\textit{av}}$ methods varies between 15 and 20 m across all three sites, with an approximate inter-method variability of $\pm 5\,\hbox {m}$. This increases to $\pm 10\,\hbox {m}$ when the river sector is included, with $z_{d}$ values as low as 5 m at the KSK site. The variability of the $\textit{RE}_{\textit{av}}$ methods in the river sector (Fig. 5a–c) is proportional to the extent of the source area that is occupied by the river, which reduces $\lambda _{p}$. Between the methods, $\textit{Bot}_{z_{d}}$ is consistently smallest and $\textit{Mac}_{z_{d}}$ is the largest for more densely packed directions.

As expected from the sensitivity analysis (Fig. 1), $\textit{Kan}_{z_{d}}$ is consistently up to 5 m larger than $\textit{Mho}_{z_{d}}$ (Fig. 5a–c). Both methods indicate $z_{d}$ $\ge 1.5H_{\textit{av}}$ for the surrounding area—a value typically used to estimate the minimum RSL depth (Roth 2000). Such high $z_{d}$ values support the contention that roughness-element height variability is important when considering the determination of $H_{\textit{RSL}}$, in addition to, for example, $H_{\textit{av}}$ and roughness-element spacing (Cheng and Castro 2002). An effective mean building height has been suggested as a more appropriate scaling parameter for $H_{\textit{RSL}}$ that incorporates building-height variability (Millward-Hopkins et al. 2011, their Eq. 21). It may also be possible to consider the influence of height variability on $H_{\textit{RSL}}$ through directly considering $\sigma _{H}$ or $H_{\textit{max}}$ (e.g. $H_{\textit{RSL}}= 2H_{\textit{av}}+\sigma _{H})$. At the KSK site, the $z_{d}$ value determined by the $\textit{RE}_{\textit{var}}$ methods is consistently of the order of the measurement height, or greater, suggesting that the flux footprint either cannot be calculated or is consistently smaller than 100 m in horizontal extent and therefore few values are reported here (Fig. 5c, f).

If the $f_\mathrm{d}$ constant used in the *RT* method is doubled (Eq. 2), the predicted $z_{d}$ value aligns reasonably well with the $z_{d}$ value estimated by the $\textit{RE}_{\textit{var}}$ methods (Fig. 5a–c, *2RT*). This suggests that if limited geometric parameters are available (i.e. only $H_{\textit{av}})$, the choice of $2\textit{RT}_{z_{d}}$ may provide a useful proxy for $z_{d}$ determined by the $\textit{RE}_{\textit{var}}$ methods in a heterogeneous mix. Assessment of the geometric parameters for each morphometric method’s respective source area indicates the magnitude of $z_{d}$ for all methods is fundamentally determined by the directional variability in $\lambda _{p}$. This includes $\textit{Mho}_{z_{d}}$ and $\textit{Kan}_{z_{d}}$, both of which are more sensitive to variability in $\lambda _{p}$, despite their direct incorporation of $\sigma _{H}$ and/or $H_{\textit{max}}$.

### Aerodynamic Roughness Length $(z_{0})$

#### $z_{0}$ Determined by Anemometric Methods

The aerodynamic roughness length determined using the EC method is a function of both observations (i.e. $\bar{u}_\mathrm{z}$ and $u_{*}$ for each 30-min observation) and the $z_{d}$ determined using the temperature and velocity variance methods. Therefore, the consistently larger $z_{d}$ determined using the temperature variance method (Fig. 5a–c) implies that the associated $z_{0}$ is consistently lower than that of the velocity variance method. For each method, the interquartile range of $z_{0}$ (Fig. 5d–f shading around lines) consistently falls within $\pm 0.25\,\hbox {m}$ from the median for each $10^{\circ }$ sector. In directions where turbulence data indicate disturbance (Sect. 4.2, Fig. 5, directions with red shading) there is an increase in $z_{0}$ because of the increased friction velocity in the same direction.

In directions without the river, the median $z_{0}$ varies between 0.25 and 3 m, tending towards the lower end of typical $z_{0}$ values reported for cities (Grimmond and Oke 1999). This is likely because the dense packing of roughness elements ($\lambda _{f}$ and $\lambda _{p}\ge 0.5$) creates a flow more characteristic of skimming than chaotic (e.g. Oke 1987).

When the flow is aligned with the river (Fig. 5d–f, between $090^{\circ }{-}120^{\circ }$ and $190^{\circ }{-}210^{\circ })$, $z_{0}$ values become smallest at the KSSW and KSS sites (as low as 0.1 m) because of flow along the smoother more homogeneous surface. This reduction is not obvious at the KSK site because of its lower siting and associated smaller source area (i.e. these measurements tend not to be affected by the river) (Sect. 7). At the KSSW site a reduction in $z_{0}$ to 0.25 m also occurs when the flow is aligned with the adjacent Strand street canyon ($060^{\circ }$, Fig. 2), because of the reduction of drag as flow is channelled along the canyon. The effect of the channelling is not observed at the KSK site because of its lower and more southerly siting, nor at the KSS site because of the microscale anthropogenic heat and moisture source in the same direction (Sect. 4.2).

#### $z_{0}$ Determined by Morphometric Methods

The morphometric methods (except for the *Mho* method) have relative peaks in $z_{0}$ at the edges of the river sector (Fig. 5 blue shading) similar to where the anemometric $z_{0}$ becomes lowest (Sect. 5.2.1). This is because, although the majority of a source area may lack roughness elements and be smooth, the morphometric methods are responsive to the geometry calculated within the source area, which according to the morphometric method formulations generates disrupted flow. The peaks in the morphometrically-determined $z_{0}$ occur when the source area falls upon both river and buildings causing $\lambda _{f}$ to be close to $\lambda _{{f-crit}}$ (Fig. 1). When most of the source area is river, $\lambda _{f}$ becomes smallest $(\lambda _{f }=0.2)$. Here, the *Mho* method indicates the highest $z_{0}$ because the maximum $\textit{Mho}_{{z}_{0}}$ occurs at these smaller $\lambda _\mathrm{f}$ values (Fig. 1).

All morphometric methods indicate increased roughness to the north of the sites, in response to increased roughness-element height ($H_{\textit{av}}$ up to 30 m). The variable surface morphology implies that inter-method variability is largest in these directions, varying between 1 and 4 m. In comparison, inter-method variability is least in the river sector (1.0–3.5 m), associated with the most consistent surface morphology. The directional variability of $z_{0}$ is primarily a function of $\lambda _{f}$ for all methods (except the *RT* method). The $\lambda _{f}$ value varies between 0.2 and 0.8 with wind direction, and the greater sensitivity of $\textit{Bot}_{{z}_{0}}$ and $\textit{Mac}_{{z}_{0}}$ to $\lambda _\mathrm{f}$ (Fig. 1), implies they vary most with direction. $\textit{Bot}_{{z}_{0}}$ is consistently 2 m larger than all other morphometric methods because of its more pronounced peak of $z_{0}$ (Fig. 1). In comparison, $\textit{Mac}_{{z}_{0}}$ tends to be lowest, especially where there is a greater frontal area index of roughness elements (e.g. $240^{\circ }-300^{\circ }$ where $\lambda _{f}\ge ~0.5$) because of its comparatively steep reduction of $z_{0}$ at higher $\lambda _{f}$ (e.g. Fig. 1).

The inclusion of $\textit{Mac}_{{z}_{0}}$ in $\textit{Kan}_{z_{0}}$ means that they vary similarly with direction. However, $\textit{Kan}_{z_{0}}$ tends to be 1–2 m larger than $\textit{Mac}_{{z}_{0}}$ in directions with higher frontal area, as the former does not have the steep drop off found in $\textit{Mac}_{{z}_{0}}$ at higher $\lambda _{f}$ (e.g. $240^{\circ }-300^{\circ }$ at the KSSW and KSS sites). An increasingly smaller source area occurs as the $\textit{RE}_{\textit{var}}$ method values of $z_{d}$ become similar to the measurement height at the KSK site. This explains the spread and lack of calculated $\textit{Kan}_{z_{0}}$ and $\textit{Mho}_{z_{0}}$ here (Fig. 5f).

### Comparison Between Anemometric and Morphometric Aerodynamic Parameters

**Fig. 6:**
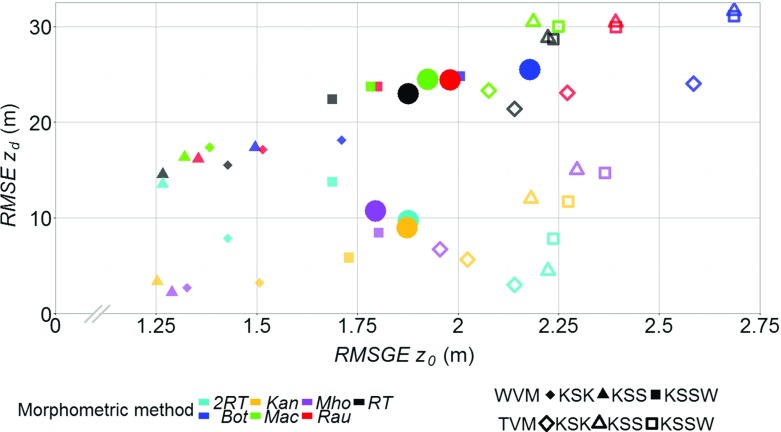
Root-mean-square error (*RMSE*) analysis of the aerodynamic parameters determined using the morphometric and anemometric methods for each 30-min period of observations at each site (*smaller symbols*) and for all observations (*larger symbols*). The *RMSE* value for $z_{d}$ is plotted against root-mean-square geometric error (*RMSGE*) for $z_{0}$ (Eq. 24). The $z_{d}$ is for unstable conditions ($-6.2 \le z'/L \le -0.05$ with $z_{d}$ in $z'/L$ for each morphometric method) and $z_{0}$ is for neutral conditions ($|z'/L|\le 0.05$, with $z_{d}$ in $z'/L$ for each morphometric method). See Tables 1 and 2 for method abbreviations

Application of the anemometric and morphometric methods at the London sites indicates that no individual value or method is optimum for aerodynamic parameter determination. Furthermore, the variability within and between the anemometric methods suggest it is not straightforward to use these as a basis for assessing morphometric methods. Therefore, the morphometric and anemometric $z_{d}$ are compared using the root-mean-squared error $(\textit{RMSE}_{z_{d}})$. For comparison of $z_{0}$ the logarithmic influence (e.g. Eq. 1) is accounted for by using the root-mean-squared geometric error $({\textit{RMSGE}}_{z_{0}})$ (Jachner et al. 2007)

24 $$\begin{aligned} \textit{RMSGE}_{z_{0}}=\text {exp}\left[ \frac{\sum \nolimits _{i=1}^n {\ln }{(A_{{z}_{0}}/M_{{z}_{0}})}^{2} }{n} \right] ^{0.5}, \end{aligned}$$

where $\textit{Az}_{0}$ and $\textit{Mz}_{0}$ are the anemometric and morphometrically determined $z_{0}$, respectively. The ${{ RMSE}}_{z_{d}}$ and ${\textit{RMSGE}}_{z_{0}}$ values between each morphometric and anemometric method at each site are plotted against each other in Fig. 6 (smaller symbols), with the larger circles representing the values for all observations.

Errors across the sites range between 2.25 and 31.4 m for zero-plane displacement and 1.25–2.7 m for roughness length (Fig. 6). For $z_{d}$, similarity between the anemometric methods and the $\textit{RE}_{\textit{var}}$ morphometric methods (Figs. 5, 6), suggests $z_{d}>H_{\textit{av}}$ in the surrounding area (20 m, Table 3). Use of the *Kan*, *Mho* and *2RT* methods results in the lowest ${{ RMSE}}_{z_{d}}$ across all observations (approximately 10 m), in comparison to the $\textit{RE}_{\textit{av}}$ methods that have ${{ RMSE}}_{z_{d}}$ $=25\,\hbox {m}$ (Fig. 6, large circles). The morphometrically-determined $z_{0}$ is consistently greater than the anemometric $z_{0}$ (Fig. 5d–f), which is more obvious for the temperature variance method $({\textit{RMSGE}}_{z_{0}}$ up to 2.70 m) than the wind variance method $({\textit{RMSGE}}_{z_{0}}$ of up to 2 m) (Fig. 6). No individual morphometric method calculates $z_{0}$ that is consistently similar to the anemometric methods, with ${\textit{RMSGE}}_{z_{0}}$ values for all observations ranging between 1.75 and 2 m (Fig. 6, circles). However, $\textit{Bot}_{z_{0}}$ deviates the furthest from observations $({\textit{RMSGE}}_{z_{0}}>2.2\,\hbox {m})$ given its considerably larger magnitude (Fig. 5d–f).

Both aerodynamic parameters $$(z_{d}$$ and $$z_{0})$$ are required for use in the logarithmic wind law. The difference in $$z_{d}$$ between the $$\textit{RE}_{\textit{var}}$$ and $$\textit{RE}_{\textit{av}}$$ methods is not compensated for in their respective $$z_{0}$$ values. Therefore, $$z_{d}$$ and $$z_{0}$$ determined by the $$\textit{RE}_{\textit{var}}$$ methods are consistently almost twice that of the $$\textit{RE}_{\textit{av}}$$ methods. The *2RT* method $$(2\textit{RT}_{z_\mathrm{d}}+2\textit{RT}_{z_{0}})$$ is closest to observations for both $$z_{d}$$ and $$z_{0}$$, despite being a simple method to bring the *RT* method in line with the $$\textit{RE}_{\mathrm{var}}$$ methods. In contrast, the *Bot* method is consistently furthest from observations for both aerodynamic parameters.

### Reference-Based Approach

**Fig. 7:**
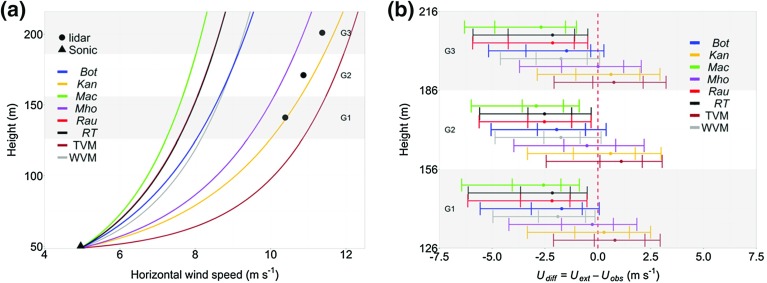
Variation of mean hourly wind speed with height (33 profiles, see text for selection): **a** mean of extrapolated profiles (Eq. 25) with locally determined aerodynamic parameters from the KSS site (*triangle*) and each 30-m lidar gate (*points*). **b** Median (*points*) difference $(U_{\textit{diff}})$ between observed $(U_{\textit{obs}})$ and extrapolated $(U_{\textit{ext}})$ wind speeds at 30-m lidar gates. Whiskers are the 5th, 25th, 75th and 95th percentiles. Gates are shaded G1–G3 $(\hbox {G}1=126-156\,\hbox {m},\,\hbox {mid-point}=141\,\hbox {m})$. See Tables 1 and 2 for method abbreviations

Aerodynamic parameters from numerous field studies using observations and morphometric methods (the $\textit{RE}_{\textit{av}}$ methods only) informed Grimmond and Oke’s (1999, their Table 6 and Fig. 7) synthesis, which is complemented with photography for application. Use of a reference-based approach to determine aerodynamic parameters at the KCL sites indicates only that $z_{d}>7\,\hbox {m}$ and $z_{0}>0.8\,\hbox {m}$ for all directions. This demonstrates the limitations of using reference-based approaches in complex urban areas, as they offer a broad range of values. In addition, the reference-based approach does not have sufficient detail to resolve the directional variability in $z_{d}$ and $z_{0}$ with local features, such as the channelling of wind flow along the River which lowered $z_{0}$ determined from observations (Sect. 5.2.1). The variability in both land cover and roughness-element height are only coarsely considered in reference classes. In addition, use of aerial photography remains subjective—for example ‘high’ and ‘high-rise’ categories (Grimmond and Oke 1999 their Fig. 7) both occur in the vicinity of the KCL sites, so selection may be inconsistent.

## Independent Method Assessment—Wind-Speed Profile Extrapolation

With an observed wind speed $(\bar{u}_{\textit{ref}})$ at a reference height $(z_{\textit{ref}})$ during neutral conditions, locally determined aerodynamic parameters can be used to estimate the wind speed ($\bar{u}_\mathrm{z})$ at a second height (*z*) using the logarithmic wind law (e.g. Wieringa 1993; Verkaik 2000)

25 $$\begin{aligned} \bar{u}_\mathrm{z}=\bar{u}_{ref}\dfrac{\ln \left\{ \dfrac{\left( z - z_{d} \right) }{z_{0}} \right\} }{\ln \left\{ \dfrac{\left( z_{ref} - z_{d} \right) }{z_{0}} \right\} } . \end{aligned}$$

The different methods to determine $z_{d}$ and $z_{0}$ are independently assessed through comparing wind speeds estimated using the logarithmic law by each method (Eq. 25) to wind-speed profiles observed with Doppler lidar (Fig. 3, $\hbox {L}_{\mathrm{1}}$). For the comparison, the lidar is located at the KSSW site location (Sect. 4.2). Therefore, observations from the KSS site (45 m east of the KSSW site, Fig. 2) provide $\bar{u}_{ref}\,(z_{\textit{ref}}= 48.9~\hbox {m})$ and other variables (Eq. 25). Hourly data are used to ensure acceptable errors in the lidar data (Lane et al. 2013). The wind speed for each method is calculated at 1-m height intervals and then averaged over 30-m “gates” to correspond to the vertical resolution of the lidar.

Observations at a greater height have a larger source area. Identical fetch in any direction is rare in an urban area, therefore, it is likely that $z_{d}$ and $z_{0}$ should also adjust with source area. To constrain changes in $z_{d}$ and $z_{0}$ throughout the profile, as well as the likelihood of overlapping internal boundary layers from surface discontinuities (e.g. Garratt 1990), the analysis is undertaken for the most homogeneous fetch within 10 km of the KSSW site (Fig. 2). This is deemed to be the $000^{\circ }{-}045^{\circ }$ direction based upon 500-m grid squares of average ground height and the $H_{\textit{av}}$, $H_{\textit{max}}$ and $\sigma _{H}$ values of roughness elements from the surface elevation database (Lindberg and Grimmond 2011).

Outside of neutral stability, corrections are required to the logarithmic wind profile. These are based upon empirical fits to observations aloft of idealized surfaces and can vary considerably (Högström 1996). Such corrections therefore introduce a source of uncertainty into extrapolated wind speeds and given the objective to evaluate aerodynamic parameters determined by different methods, only neutral stability is considered here. To ensure wind-speed profiles are most likely for neutral stability, the highest (upper quartile) wind speeds are used (Drew et al. 2013). Regression between the inverse of the Obukhov length (*1/L*) and wind speed measured at the KSS site for the same times confirms the tendency of the stability parameter $z'/L$ towards zero (neutral) as wind speeds increase. To ensure the depth of the urban boundary layer is sufficient, analysis is restricted to the lowest 200 m of daytime (0900–1700 h) profiles so the logarithmic wind law is appropriate (Cook 1997; Tieleman 2008; Li et al. 2010; Drew et al. 2013). After filtering the lidar data, 33 profiles are available from the $000^{\circ }-045^{\circ }$ sector with upper quartile wind speeds. Data are analyzed from the lowest three gates (mid-points: 141, 171 and 201 m).

The mean observed wind speeds in each 30-m gate are 10.4, 10.9 and $11.4\hbox { m s}^{-1}$ (lowest to highest, Fig. 7a). These are most similar to the greater wind speeds extrapolated using aerodynamic parameters from the *Kan*, *Mho* and temperature variance methods (Fig. 7a). Both $z_{d}$ and $z_{0}$ are free parameters in Eq. 25, therefore two different pairs of values can predict the same wind speed aloft. However, the comparatively lower $z_{d}$ of the $\textit{RE}_{\textit{av}}$ methods and lack of compensation for this in $z_{0}$ means that their extrapolated wind speeds are less than those from both the $\textit{RE}_{\textit{var}}$ methods and observations (Fig. 7).

The differences $(U_{\textit{diff}})$ between wind speeds extrapolated using the different methods and wind speeds observed by the lidar (for each of the 33 profiles compared) are summarized in Fig. 7b. Over 95% of observed wind speeds are underestimated by the $\textit{RE}_{\textit{av}}$ methods, with median underestimation between 1.5 a and $2.9\hbox { m s}^{-1}$ (Fig. 7b). The higher extrapolated wind speeds using the $\textit{RE}_{\textit{var}}$ methods have a median $U_{\mathrm{diff}}<0.6\hbox { m s}^{-1}$ for all three lidar gates, which is within 6% of the mean observed wind speed. In addition, wind speeds extrapolated using the $\textit{RE}_{\textit{var}}$ methods most resemble the distribution of observed wind speeds, tending to evenly underestimate or overestimate observations (approximately 50% of cases respectively). The temperature variance method’s largest $z_{d}$ and smallest $z_{0}$ produce a consistent overestimate in the wind speed (75% of cases), however it still shows a median $U_{\mathrm{diff}} <1.1\hbox { m s}^{-1}$ for all gates (Fig. 7b).

Results suggest that if high wind speeds are of concern, aerodynamic parameters determined using the *Mho*, *Kan* or temperature variance methods may be the most appropriate methods to estimate the neutral vertical profile of wind speed. No relation is observed between the individual $U_{\textit{diff}}$ values and either meteorological conditions (e.g. *L*, $\bar{u}_{\textit{ref}}$, $u_{*}$) or the time of day. However, there are other potential reasons why differences in wind speed occur. Although the most homogeneous direction was selected $(000^{\circ }{-}045^{\circ })$, the difference in source area between the sensor used for extrapolation $(z=48.9\,\hbox {m})$ and lidar $(z=126{-}216\,\hbox {m})$ implies that the flow is likely in equilibrium with different upwind surfaces. Accounting for the changes in upwind surface morphology may therefore improve wind-speed estimation. The concept of a blending height $(z_{b})$ above which the wind-speed profile is respond to an entire heterogeneous surface (Grimmond and Oke 1999; Roth 2000; Barlow 2014) may support this hypothesis, however there is uncertainty in the determination of $z_{b}$ (Grimmond and Oke 1999; Grimmond et al. 2004; Barlow 2014). A further consideration is the depth of the ISL and therefore the theoretical validity of the logarithmic wind-speed profile to the heights assessed. However, the comparison was limited to daytime profiles below 216 m and the individual observed wind-speed profiles (Fig. 7a) indicate profiles compared are logarithmic in nature.

**Fig. 8:**
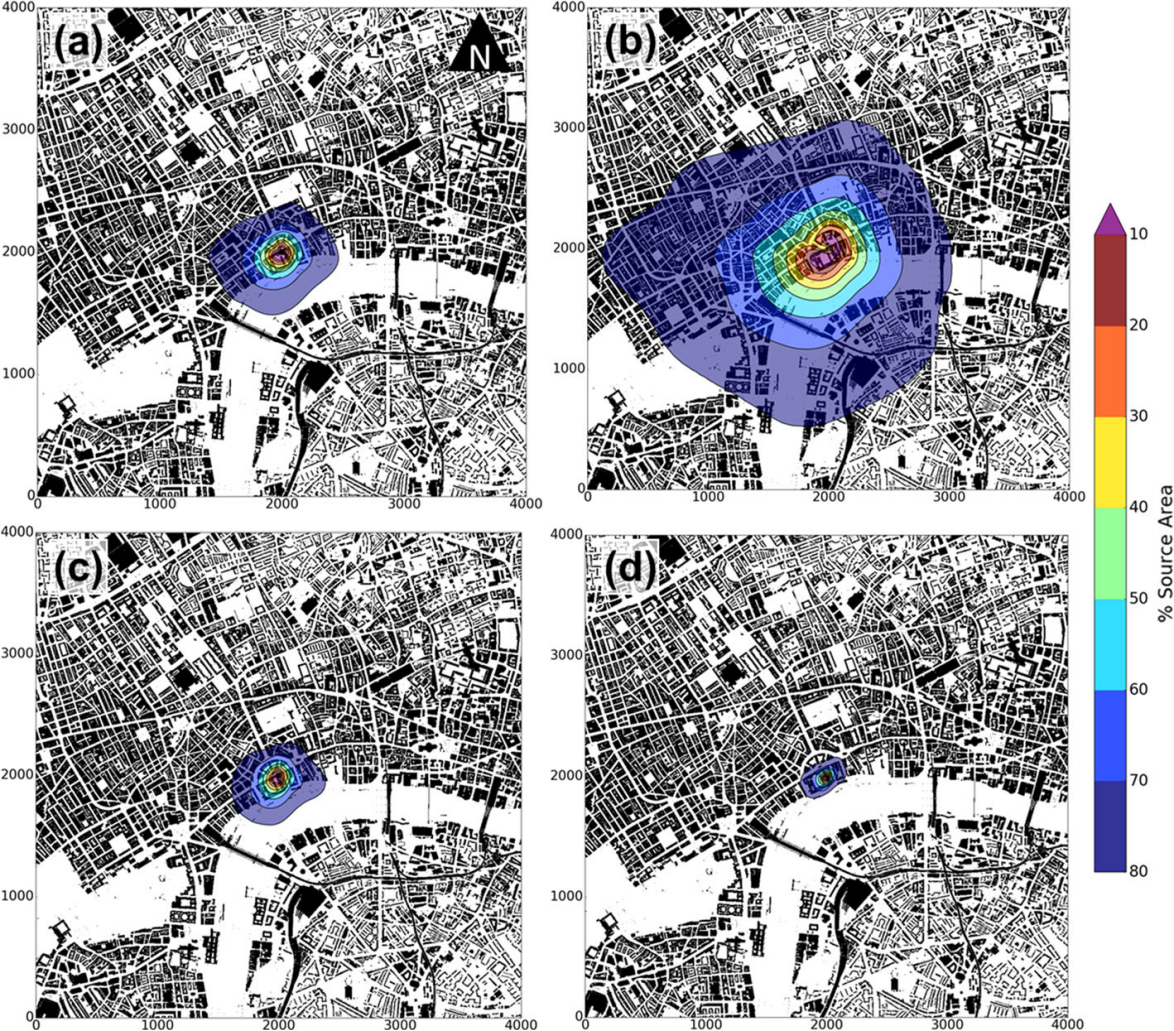
Integrated annual 80% source areas at the: **a, b** KSSW, **c** KSS and **d** KSK sites, normalized for the observation period (Table 3, from 30-min averaged observations, Sect. 3.2.2). Source areas are determined using the Kormann and Meixner (2001) analytical footprint model with aerodynamic parameters from the **a, c, d** *Mho* and **b** *Mac* morphometric methods. Cumulative source areas are shaded with 10% contours demarcated (*black lines*). Map units are metres

## Source-Area Modelling Using the Morphometric Methods

The EC turbulent flux source area is a function of the aerodynamic parameters and meteorological conditions. The surface characteristics within the source area of an EC measurement are of interest, not only for explaining the flux partitioning (Kotthaus and Grimmond 2014b) and $\hbox {CO}_{{2}}$ exchange (Ward et al. 2015), but also for determination of aerodynamic parameters themselves (which is why the iterative methodology is used, Sect. 4.3.3). To assess the impact of the aerodynamic parameters determined by each morphometric method on the modelled source area, a footprint climatology for each method is generated at each site. The sum of all 80% cumulative weight source areas (Sect. 4.3.3) for each 30-min mean observation is normalized by the total sum of weights. As different years are analyzed (2014 at the KSSW site; 2011 at the KSS and KSK sites) direct comparison is not undertaken. However, the lower the height of the sensor, the smaller the modelled source area (i.e. KSSW, KSS to KSK site—Fig. 8). In addition, the source-area climatology is biased towards the dominant south-westerly wind direction. The greatest wind speeds from the south-west, as well as more frequent neutral conditions, means the source areas also extend furthest upwind in this direction.

**Table 4:** Characteristics of the annual source area (80%) for each site (Fig. 8): (a) Geometric parameters and (b) Surface cover. See Tables 1 and 3 for measurement time periods and method/ geometry abbreviations

Site	Morphometric method	$H_{\textit{av}}$ (m)	$\lambda _{p}$	$\lambda _{f}$	$H_{\textit{max}}$ (m)	$\sigma _{H}$ (m)
(a) Geometric parameters: median (*min*, *max*)						
KSSW	*Mho*	23.01	0.42	0.49	52.11	9.50
		(9.80, 30.14)	(0.21,.90)	(0.12, 3.01)	(31.97, 184.73)	(4.71, 16.29)
	*Mac*	21.30	0.40	0.43	77.80	10.21
		(9.30. 29.93)	(0.16, 0.79)	(0.04, 2.71)	(32.64 , 184.73)	(4.53, 17.63)
KSS	*Mho*	23.41	0.44	0.48	46.10	9.22
		(10.76, 30.74)	(0.25, 0.84)	(0.11, 2.80)	(34.42, 184.73)	(5.67, 13.96)
KSK	*Mho*	23.38	0.55	0.63	39.51	8.48
		(18.38, 29.76)	(0.32, 0.99)	(0.19, 2.27)	(28.60, 184.73)	(3.66, 13.59)

Site		Built	Paved	Grass	Trees and shrubs	Water
(b) Surface Cover (%) for 80% source area						
KSSW	*Mho*	42	48	3	1	6
	*Mac*	40	39	4	3	14
KSS	*Mho*	45	48	2	1	4
KSK	*Mho*	57	42	1	0	0

The surface characteristics weighted by the footprint climatology (Fig. 8, Table 4) are different to those of the unweighted surrounding 1-km radius (Table 3). The similar measurement heights at the KSSW and KSS sites implies that their footprint climatology characteristics are similar. In comparison, the lower siting of the KSK site produces a smaller source area (Fig. 8d), which is predominantly built and paved, with only 0.7% water. A wide range of geometric parameters occur in the source areas (Table 4a), which modifies the ratio of the measurement height to roughness-element heights. The median $H_{\textit{av}}$ for all sites is approximately 23 m and roughness-element height varies between 9.2 and 9.5 m (median $\sigma _{H}$). The smallest $H_{\textit{av}}$ recorded is 10 m, in which case the measurement height $=5H_{\textit{av}}$ and well above the RSL (Sect. 2.1). However, some source areas have $H_{\textit{av}}= 30\,\hbox {m}$, in which case measurements are at $z= 1.67H_{\textit{av}}$ and therefore more likely influenced by roughness-element wakes.

The source areas modelled using the $\textit{RE}_{\textit{av}}$ methods are larger than the $\textit{RE}_{\textit{var}}$ methods because the greater zero-plane displacement of the latter leads to a smaller effective height of the measurements. For example, $\textit{Mho}_{{z}_\mathrm{d}}$ is typically twice $\textit{Mac}_{{z}_{d}}$ and a comparison of the source areas modelled at the KSSW site using each respective method demonstrates this difference (Fig. 8a, b). The upwind distance contributing to the 80% cumulative source area is consistently over three times further in all directions for the *Mac* method. This influences the surface characteristics that are determined for the source area. For example, the parks to the south-west of the sites (Sect. 4.1) are not within the *Mho* method source area, but fall within the 80% of the *Mac* method, explaining the larger proportion of vegetated land cover (grass and trees) using the latter (Table 4b). Geometric parameters are also influenced, which subsequently influence morphometrically-determined aerodynamic parameters. For example, the larger source area modelled using aerodynamic parameters from the *Mac* method gives a relatively larger $H_{\textit{max}}$, $\sigma _{H},\lambda _{p},\lambda _{f}$ and lower $H_{\textit{av}}$ than within the *Mho* method source area (Table 4a).

## Conclusions

Morphometric and anemometric analysis of aerodynamic parameters for three adjacent sites in Central London give estimates of zero-plane displacement $(z_{d})$ between 5 and 45 m and aerodynamic roughness length $(z_{0})$ between 0.1 and 5 m. A source-area footprint model (Kormann and Meixner 2001) is used to apply the morphometric methods in an iterative procedure. Although a first-order estimate of $z_{d}$ and $z_{\mathrm{0}}$ is required, the final $z_{d}$ and $z_{0}$ values are similar, independent of the initial estimation. This conclusion is true for another source-area model (Kljun et al. 2015), indicating that an iterative procedure removes the need for initial site specific values. This saves time and also ensures more appropriate values of the aerodynamic parameters and source area dimensions.

Two methods that rely on surface-layer scaling during unstable conditions are used to determine $z_{d}$ from observations (Rotach 1994; Toda and Sugita 2003). The methods, not obviously sensitive to the initial $z_{d}$ used to define stability, agree that $z_{d}$ is larger than the average roughness-element height $(H_{\textit{av}})$ in the surrounding 1-km fetch. Although this conclusion is supported by the literature, previously these values have been considered unreasonably large (Grimmond et al. 1998, 2002; Feigenwinter et al. 1999; Kanda et al. 2002; Tsuang et al. 2003; Christen 2005; Chang and Huynh 2007).

Morphometric methods to determine $z_{d}$ can be split into two types based on the attributes of roughness-element height used, i.e. the average height $(\textit{RE}_{\textit{av}})$ or the variability/ maximum height $(\textit{RE}_{\textit{var}})$. The zero-plane displacement determined by the $\textit{RE}_{\textit{var}}$ methods is consistently larger than $H_{\textit{av}}$ and twice the magnitude of that from the $\textit{RE}_{\textit{av}}$ methods, which is approximately $0.7H_{\textit{av}}$. A simple doubling of $z_{d}$ determined by a rule-of-thumb morphometric method that is based only upon average roughness-element height, brought values more in line with the $z_{d}$ values determined using the $\textit{RE}_{\textit{var}}$ methods.

There is agreement between anemometric methods and the morphometric methods which consider height variability, that $z_{d}$ is larger than $H_{\textit{av}}$. This conclusion is supported by numerical and physical experiments (e.g. Jiang et al. 2008; Hagishima et al. 2009; Zaki et al. 2011; Millward-Hopkins et al. 2011; Tanaka et al. 2011; Kanda et al. 2013) indicating the taller roughness elements in a heterogeneous mix exert a disproportionate amount of drag on the flow (Xie et al. 2008; Mohammad et al. 2015b) lifting the drag-profile centroid (Jackson 1981) above $z=H_{\textit{av}}$. The results verify Kanda et al.’s (2013) proposition that the maximum height $(H_{\textit{max}})$ is a more suitable scaling parameter for $z_{d}$ and the standard deviation of the roughness-element height $(\sigma _{H})$ (also used by Millward-Hopkins et al. 2011) is useful to parametrize roughness-element height heterogeneity. This conclusion has implications for the interpretation of output from anemometers (and potentially other meteorological sensors) in the heterogeneous urban environment. Sensors may need to be located higher above roughness elements to provide a local-scale (or neighbourhood), rather than microscale, measurement.

Morphometric-based $z_{0}$ values are consistently larger than the anemometric $z_{0}$ by 2–3 m. Although the two classes of morphometric methods ($\textit{RE}_{\textit{av}}$ and $\textit{RE}_{\textit{var}}$) do not demonstrate an obvious difference, root-mean-square error analysis demonstrates the $\textit{RE}_{\textit{var}}$ methods are most similar to observations. Individual $\textit{RE}_{\textit{av}}$ methods consistently result in the largest (Bottema and Mestayer 1998) and smallest (Macdonald et al. 1998) $z_{0}$ values.

The ability of each method to correctly estimate wind speed with height is assessed using locally determined aerodynamic parameters and the logarithmic wind law. Wind speeds observed with Doppler lidar (up to 200 m above the canopy) are underestimated with the $\textit{RE}_{\textit{av}}$ morphometric methods (median underestimation: $1.5-2.9\hbox { m s}^{-1}$ for average wind speeds: $10.4-11.4\,\hbox {m s}^{-1})$. Whereas, the larger $z_{d}$ determined using the $\textit{RE}_{\textit{var}}$ methods provides similar results to the observations (median differences $<0.62\,\hbox {m s}^{-1}$), demonstrating the importance of considering roughness-element height heterogeneity when estimating the wind-speed profile.

The modelled eddy-covariance source area is typically a third (or smaller) of the size when $\textit{RE}_{\textit{var}}$ methods are used, as the effective measurement height (i.e. with $z_{d}$ accounted for) tends to be half that of the $\textit{RE}_{\textit{av}}$ methods. This has implications for land-cover and geometric parameters determined for a source area and their subsequent uses.

The tools for morphometric determination of $z_{d}$ and $z_{0}$ (including the two footprint models used) are available in the Urban Multi-Scale Environmental Predictor (UMEP, http://www.urban-climate.net/umep/UMEP, Lindberg et al. 2016), which is an extension to the open source geographical information software QGIS.

## Appendix

See Table 5.

**Table 5:** Methods in the literature (ordered by date) used to calculate the zero-plane displacement $(z_{d})$ and aerodynamic roughness length $(z_{0})$ from (a) morphometric and (b) anemometric data with the stability conditions required

Reference	Background to method		
(a) Morphometric method			
Kutzbach (1961)	Bushel baskets on frozen lake		
Lettau (1969)	Wind-tunnel and Kutzbach (1961) data		
Fang and Sill (1992)	Wind-tunnel experiments		
Kondo and Yamazawa (1986)	Two urban districts of Japan		
Counihan (1971)	Regular arrays of cubic blocks wind-tunnel data		
Theurer (1993)	Field experiments and wind-tunnel data		
*Raupach (1994) *Rau*	Wind-tunnel and rough vegetated surface data		
Bottema (1995, 1997)	Regular, staggered and varying density array of blocks wind-tunnel data		
*Bottema and Mestayer (1998) *Bot*	Simplification of Bottema (1995, 1997) for use in urban areas		
*Macdonald et al. (1998) *Mac*	From fundamental principles and wind-tunnel data (Hall et al. (1996))		
*Grimmond and Oke (1999) *RT*	Rule-of-thumb from synthesis of wind-tunnel and field results		
Kastner-Klein and Rotach (2004)	Scaled model of Nantes, France, wind-tunnel data		
Nakayama et al. (2011)	Large-eddy simulation (LES) using various building arrays		
Millward-Hopkins et al. (2011)	Quasi-empirical modelling and development of previous models		
*Millward-Hopkins et al. (2011) *Mho*	Simplification of Millward-Hopkins et al. (2011) using elevation data from Leeds, UK		
*Kanda et al. (2013) *Kan*	LES with explicitly resolved buildings in Tokyo, Japan		

Method	Reference	Anemometric data required	Stability
(b) Anemometric method			
$$z_{0}$$			
Standard deviation	Beljaars (1987)	Single level, fast or slow response	Neutral
*Eddy covariance	Grimmond et al. (1998) EC	Single level, fast response	Neutral
$$z_{d}$$			
*Temperature variance	Rotach (1994) TVM	Single level, fast response	Unstable
*Vertical wind variance	Toda and Sugita (2003) WVM	Single level, fast response	Unstable
Spectral	Christen (2005)	Single level, fast response	Neutral
$z_{0}$ and $z_{d}$			
Profile	Lettau (1957)	Profile, fast or slow response	Neutral
Regressed profile	Schaudt (1998)	Profile, fast or slow response	Neutral
Least-squares	Martano (2000)	Single level, fast response	All

Methods used in this study are indicated (*) and have their abbreviation used in the Reference column

## References

[CR1] Arnfield AJ (2003). Two decades of urban climate research: a review of turbulence, exchanges of energy and water, and the urban heat island. Int J Climatol.

[CR2] Barlow JF (2014). Progress in observing and modelling the urban boundary layer. Urban Clim.

[CR3] Barlow JF, Dobre A, Smalley R, Arnold S, Tomlin A, Belcher SE (2009). Referencing of street-level flows measured during the DAPPLE 2004 campaign. Atmos Environ.

[CR4] Bates DM, Watts DG (1988) Nonlinear regression: iterative estimation and linear approximations. In: Bates DM, Watts DG (eds) Nonlinear regression analysis and its applications. Wiley, Hoboken, 365 pp

[CR5] Beljaars AC (1987) The measurement of gustiness at routine wind stations: a review. R Neth Meteorol Inst Sci Rep WR-87-11 (WMO Instr. Meth. Obs. Rep. 31), 50 pp

[CR6] Björkegren A, Grimmond C, Kotthaus S, Malamud B (2015). CO$$_2$$ emission estimation in the urban environment: measurement of the CO$$_2$$ storage term. Atmos Environ.

[CR7] Bottema M (1995) Aerodynamic roughness parameters for homogenous building groups—part 2: results. Ecole Centrale de Nantes, France Document SUB-MESO #23

[CR8] Bottema M (1997). Urban roughness modelling in relation to pollutant dispersion. Atmos Environ.

[CR9] Bottema M, Mestayer PG (1998). Urban roughness mapping-validation techniques and some first results. J Wind Eng Ind Aerodyn.

[CR10] Britter R, Hanna S (2003). Flow and dispersion in urban areas. Annu Rev Fluid Mech.

[CR11] Chang S, Huynh G (2007) A comparison of roughness parameters for Oklahoma City from different evaluation methods. AMS 7th symposium on the Urban environment 9.2. https://ams.confex.com/ams/7Coastal7Urban/techprogram/paper_126674.htm. Accessed 1 April 2017

[CR12] Cheng H, Castro IP (2002). Near wall flow over urban-like roughness. Boundary-Layer Meteorol.

[CR13] Cheng H, Hayden P, Robins A, Castro I (2007). Flow over cube arrays of different packing densities. J Wind Eng Ind Aerodyn.

[CR14] Christen A (2005) Atmospheric turbulence and surface energy exchange in urban environments: results from the Basel Urban Boundary Layer Experiment (BUBBLE). Atmospheric turbulence and surface energy exchange in urban environments: results from the Basel Urban Boundary Layer Experiment (BUBBLE), Doctoral thesis, Department of Science, University of Basel, Switzerland

[CR15] Claus J, Coceal O, Thomas TG, Branford S, Belcher S, Castro IP (2012). Wind-direction effects on urban-type flows. Boundary-Layer Meteorol.

[CR16] Cook NJ (1997). The Deaves and Harris ABL model applied to heterogeneous terrain. J Wind Eng Ind Aerodyn.

[CR17] Counihan J (1971). Wind tunnel determination of the roughness length as a function of the fetch and the roughness density of three-dimensional roughness elements. Atmos Environ.

[CR18] De Bruin H, Verhoef A (1999) Reply to the Comments on ‘a new Method to Determine the Zero-Plane Displacement’, by Zhang and Park. Boundary-Layer Meteorol 91:141–143

[CR19] Deaves D, Harris R (1978) A mathematical model of the structure of strong winds. Construction Industry Research and Information Association Report number 76, London, England

[CR20] Drew D, Barlow J, Cockerill T (2013). Estimating the potential yield of small wind turbines in urban areas: a case study for Greater London, UK. J Wind Eng Ind Aerodyn.

[CR21] Emeis S, Baumann-Stanzer K, Piringer M, Kallistratova M, Kouznetsov R, Yushkov V (2007). Wind and turbulence in the urban boundary layer-analysis from acoustic remote sensing data and fit to analytical relations. Meteorol Z.

[CR22] Evans S (2009). 3D cities and numerical weather prediction models: an overview of the methods used in the LUCID project.

[CR23] Fang C, Sill B (1992). Aerodynamic roughness length: correlation with roughness elements. J Wind Eng Ind Aerodyn.

[CR24] Feigenwinter C, Vogt R, Parlow E (1999). Vertical structure of selected turbulence characteristics above an urban canopy. Theor Appl Climatol.

[CR25] Fernando H (2010). Fluid dynamics of urban atmospheres in complex terrain. Annu Rev Fluid Mech.

[CR26] Foken T, Wichura B (1996). Tools for quality assessment of surface-based flux measurements. Agric For Meteorol.

[CR27] Garratt J (1990). The internal boundary layer—a review. Boundary-Layer Meteorol.

[CR28] Giometto M, Christen A, Meneveau C, Fang J, Krafczyk M, Parlange M (2016). Spatial characteristics of roughness sublayer mean flow and turbulence over a realistic urban surface. Boundary-Layer Meteorol.

[CR29] Grimmond CSB, King TS, Roth M, Oke TR (1998). Aerodynamic roughness of urban areas derived from wind observations. Boundary-Layer Meteorol.

[CR30] Grimmond CSB, Oke TR (1999). Aerodynamic properties of urban areas derived from analysis of surface form. J Appl Meteorol.

[CR31] Grimmond CSB, Salmond J, Offerle BD, Oke TR, (2002) Local-scale surface flux measurements at a downtown site in Marseille during the ESCOMPTE field Campaign. In: Proceedings, 4th conference on Urban environment, Norfolk, USA, 20–24 May 2002, American Meteorological Society, 45 Beacon St., Boston, MA, pp 21–22

[CR32] Grimmond CSB, Salmond J, Oke TR, Offerle B, Lemonsu A (2004). Flux and turbulence measurements at a densely built-up site in Marseille: heat, mass (water and carbon dioxide), and momentum. J Geophys Res Atmos.

[CR33] Gryning S, Batchvarova E, Brümmer B, Jørgensen H, Larsen S (2007). On the extension of the wind profile over homogeneous terrain beyond the surface boundary layer. Boundary-Layer Meteorol.

[CR34] Hagishima A, Tanimoto J, Nagayama K, Meno S (2009). Aerodynamic parameters of regular arrays of rectangular blocks with various geometries. Boundary-Layer Meteorol.

[CR35] Hall D, Macdonald JR, Walker S, Spanton AM (1996) Measurements of dispersion within simulated urban arrays—a small scale wind tunnel study. BRE Client Report, CR178/96

[CR36] Högström U (1996). Review of some basic characteristics of the atmospheric surface layer. Boundary-Layer Meteorol.

[CR37] Hsieh C, Katul GG, Schieldge J, Sigmon J, Knoerr KR (1996). Estimation of momentum and heat fluxes using dissipation and flux-variance methods in the unstable surface layer. Water Resour Res.

[CR38] Jachner S, Van den Boogaart G, Petzoldt T (2007). Statistical methods for the qualitative assessment of dynamic models with time delay (R Package qualV). J Stat Softw.

[CR39] Jackson P (1981). On the displacement height in the logarithmic velocity profile. J Fluid Mech.

[CR40] Jiang D, Jiang W, Liu H, Sun J (2008). Systematic influence of different building spacing, height and layout on mean wind and turbulent characteristics within and over urban building arrays. Wind Struct.

[CR41] Kaimal JC, Finnigan JJ (1994) Atmospheric boundary layer flows: their structure and measurement. Oxford University Press, Oxford, 289 pp

[CR42] Kanda M, Moriwaki R, Roth M, Oke T (2002). Area-averaged sensible heat flux and a new method to determine zero-plane displacement length over an urban surface using scintillometry. Boundary-Layer Meteorol.

[CR43] Kanda M, Inagaki A, Miyamoto T, Gryschka M, Raasch S (2013). A new aerodynamic parametrization for real urban surfaces. Boundary-Layer Meteorol.

[CR44] Kastner-Klein P, Rotach MW (2004). Mean flow and turbulence characteristics in an urban roughness sublayer. Boundary-Layer Meteorol.

[CR45] Kljun N, Calanca P, Rotach MW, Schmid HP (2015). A simple two-dimensional parameterisation for Flux Footprint Prediction (FFP). Geosci Mod Dev.

[CR46] Kondo J, Yamazawa H (1986). Aerodynamic roughness over an inhomogeneous ground surface. Boundary-Layer Meteorol.

[CR47] Kormann R, Meixner FX (2001). An analytical footprint model for non-neutral stratification. Boundary-Layer Meteorol.

[CR48] Kotthaus S, Grimmond CSB (2012). Identification of micro-scale anthropogenic CO$$_2$$, heat and moisture sources-processing eddy covariance fluxes for a dense urban environment. Atmos Environ.

[CR49] Kotthaus S, Grimmond CSB (2014). Energy exchange in a dense urban environment—part I: temporal variability of long-term observations in central London. Urban Clim.

[CR50] Kotthaus S, Grimmond CSB (2014). Energy exchange in a dense urban environment—part II: impact of spatial heterogeneity of the surface. Urban Clim.

[CR51] Kutzbach JE (1961) Investigations of the modification of wind profiles by artificially controlled surface roughness. Msc. Thesis. Department of Meteorology, University of Wisconsin-Madison

[CR52] Lane S, Barlow JF, Wood CR (2013). An assessment of a three-beam Doppler lidar wind profiling method for use in urban areas. J Wind Eng Ind Aerodyn.

[CR53] Leclerc MY, Foken T (2014). Footprints in micrometeorology and ecology.

[CR54] Leonardi S, Castro IP (2010). Channel flow over large cube roughness: a direct numerical simulation study. J Fluid Mech.

[CR55] Lettau H (1957) Compilation of Richardson numbers, classification of profiles and determination of roughness parameters. In: Lettau HH, Davidson B (eds) Exploring the atmopshere’s 1st Mile, Pergamon Press, London, 376 pp

[CR56] Lettau H (1969). Note on aerodynamic roughness-parameter estimation on the basis of roughness-element description. J Appl Meteorol.

[CR57] Li Q, Zhi L, Hu F (2010). Boundary layer wind structure from observations on a 325 m tower. J Wind Eng Ind Aerodyn.

[CR58] Lindberg F, Grimmond CSB (2011). Nature of vegetation and building morphology characteristics across a city: influence on shadow patterns and mean radiant temperatures in London. Urban Ecosyst.

[CR59] Lindberg F, Grimmond CSB, Capel-Timms I, Chang YY, Gabey A, Huang B, Jarvi L, Kent CW, Kokkonen T, Krave N, Olofson F, Onomura S, Sun T, Tan JG, Ward HC, Xue L, zum Berge K (2016) Urban Multi-scale Environmental Predictor (UMEP) Manual. University of Reading UK, University of Gothenburg Sweden, SIMS China. http://urban-climate.net/umep/UMEP_Manual. Accessed 6 Mar 2017

[CR60] Liu G, Sun J, Jiang W (2009). Observational verification of urban surface roughness parameters derived from morphological models. Meteorol Appl.

[CR61] Macdonald R, Griffiths R, Hall D (1998). An improved method for the estimation of surface roughness of obstacle arrays. Atmos Environ.

[CR62] Macdonald R (2000). Modelling the mean velocity profile in the urban canopy layer. Boundary-Layer Meteorol.

[CR63] Martano P (2000). Estimation of surface roughness length and displacement height from single-level sonic anemometer data. J Appl Meteorol.

[CR64] Millward-Hopkins J, Tomlin A, Ma L, Ingham D, Pourkashanian M (2011). Estimating aerodynamic parameters of urban-like surfaces with heterogeneous building heights. Boundary-Layer Meteorol.

[CR65] Mohammad A, Zaki S, Hagishima A, Ali M (2015). Determination of aerodynamic parameters of urban surfaces: methods and results revisited. Theor Appl Climatol.

[CR66] Mohammad AF, Zaki SA, Ali MSM, Aya H, Razak AA, Shirakashi M, Arai N (2015). Large eddy simulation of wind pressure distribution on heterogeneous buildings in idealised urban models. Energy Proc.

[CR67] Moncrieff J, Clement R, Finnigan J, Meyers T (2004) Averaging, detrending, and filtering of eddy covariance time series. In: Lee X, Massman W, Law B (eds) Handbook of micrometeorology. Springer, Netherlands, 250 pp

[CR68] Nakayama H, Takemi T, Nagai H (2011). LES analysis of the aerodynamic surface properties for turbulent flows over building arrays with various geometries. J Appl Meteorol Climatol.

[CR69] Oke TR (1987) Boundary layer climates, 2nd edn. Wiley, New York, 435 pp

[CR70] Oke TR (2007) Siting and exposure of meteorological instruments at urban sites. In: Borrego C, Norman AL (eds) Air pollution modelling and its application XVII. Springer, Berlin, 744 pp

[CR71] Padhra A (2010) Estimating the sensitivity of urban surface drag to building morphology, Doctoral thesis, Department of Meteorology, University of Reading, England

[CR72] Peña A, Gryning S, Mann J, Hasager CB (2010). Length scales of the neutral wind profile over homogeneous terrain. J Appl Meteorol Climatol.

[CR73] Ratti C, Di Sabatino S, Britter R (2006). Urban texture analysis with image processing techniques: winds and dispersion. Theor Appl Climatol.

[CR74] Ratti C, Di Sabatino S, Britter R, Brown M, Caton F, Burian S (2002). Analysis of 3-D urban databases with respect to pollution dispersion for a number of European and American cities. Water Air Soil Pollut Focus.

[CR75] Raupach M (1994). Simplified expressions for vegetation roughness length and zero-plane displacement as functions of canopy height and area index. Boundary-Layer Meteorol.

[CR76] Raupach M, Antonia R, Rajagopalan S (1991). Rough-wall turbulent boundary layers. Appl Mech Rev.

[CR77] Rooney G (2001). Comparison of upwind land use and roughness length measured in the urban boundary layer. Boundary-Layer Meteorol.

[CR78] Rotach MW (1994). Determination of the zero plane displacement in an urban environment. Boundary-Layer Meteorol.

[CR79] Rotach MW (1999). On the influence of the urban roughness sublayer on turbulence and dispersion. Atmos Environ.

[CR80] Roth M (2000). Review of atmospheric turbulence over cities. Q J R Meteorol Soc.

[CR81] Schaudt K (1998). A new method for estimating roughness parameters and evaluating the quality of observations. J Appl Meteorol.

[CR82] Schotanus P, Nieuwstadt F, De Bruin H (1983). Temperature measurement with a sonic anemometer and its application to heat and moisture fluxes. Boundary-Layer Meteorol.

[CR83] Sedefian L (1980). On the vertical extrapolation of mean wind power density. J Appl Meteorol.

[CR84] Sorbjan Z (1989) Structure of the atmospheric boundary layer. Prentice Hall, Old Tappan, 315 pp

[CR85] Stathopoulos T (2006). Pedestrian level winds and outdoor human comfort. J Wind Eng Ind Aerodyn.

[CR86] Stewart ID (2011). A systematic review and scientific critique of methodology in modern urban heat island literature. Int J Climatol.

[CR87] Stewart ID, Oke TR (2012). Local climate zones for urban temperature studies. Bull Am Meteorol Soc.

[CR88] Tanaka S, Sugawara H, Narita K, Yokoyama H, Misaka I, Matsushima D (2011). Zero-plane displacement height in a highly built-up area of Tokyo. Sola.

[CR89] Tennekes H (1973). The logarithmic wind profile. J Atmos Sci.

[CR90] Theurer W (1993) Dispersion of ground level emissions in complex built-up areas, Doctoral thesis, Department of Architecture, University of Karlsruhe, Germany

[CR91] Tieleman HW (2008). Strong wind observations in the atmospheric surface layer. J Wind Eng Ind Aerodyn.

[CR92] Tillman J (1972). The indirect determination of stability, heat and momentum fluxes in the atmospheric boundary layer from simple scalar variables during dry unstable conditions. J Appl Meteorol.

[CR93] Toda M, Sugita M (2003). Single level turbulence measurements to determine roughness parameters of complex terrain. J Geophys Res Atmos.

[CR94] Tominaga Y, Stathopoulos T (2013). CFD simulation of near-field pollutant dispersion in the urban environment: a review of current modeling techniques. Atmos Environ.

[CR95] Tsuang B, Tsai J, Lin M, Chen C (2003). Determining aerodynamic roughness using tethersonde and heat flux measurements in an urban area over a complex terrain. Atmos Environ.

[CR96] United Nations Publications (2014). World urbanization prospects 2014: highlights.

[CR97] Van Dijk A, Moene A, De Bruin H, Meteorology and Air Quality Group (2004) The principles of surface flux physics: theory, practice and description of the ECPACK library. Wageningen University, Wageningen, 99 pp

[CR98] Verkaik J (2000). Evaluation of two gustiness models for exposure correction calculations. J Appl Meteorol.

[CR99] Walker GR, Mason MS, Crompton RP, Musulin RT (2016). Application of insurance modelling tools to climate change adaptation decision-making relating to the built environment. Struct Infrastruct Eng.

[CR100] Ward H, Kotthaus S, Grimmond C, Bjorkegren A, Wilkinson M, Morrison W, Evans J, Morison J, Iamarino M (2015). Effects of urban density on carbon dioxide exchanges: observations of dense Urban, Suburban and Woodland areas of Southern England. Environ Pollut.

[CR101] Wieringa J, Davenport AJ, Grimmond CSB, Oke TR (2001) New revision of Davenport roughness classification. In: Proceedings of 3EACWE, Eindhoven, The Netherlands, pp 285–292

[CR102] Wieringa J (1993). Representative roughness parameters for homogeneous terrain. Boundary-Layer Meteorol.

[CR103] Wyngaard J, Coté O, Izumi Y (1971). Local free convection, similarity, and the budgets of shear stress and heat flux. J Atmos Sci.

[CR104] Xie Z, Coceal O, Castro IP (2008). Large-eddy simulation of flows over random urban-like obstacles. Boundary-Layer Meteorol.

[CR105] Zaki SA, Hagishima A, Tanimoto J, Ikegaya N (2011). Aerodynamic parameters of urban building arrays with random geometries. Boundary-Layer Meteorol.

